# A GATA2-CDC6 axis modulates androgen receptor blockade-induced senescence in prostate cancer

**DOI:** 10.1186/s13046-023-02769-z

**Published:** 2023-07-29

**Authors:** Ioanna Mourkioti, Aikaterini Polyzou, Dimitris Veroutis, George Theocharous, Nefeli Lagopati, Emanuela Gentile, Vasiliki Stravokefalou, Dimitris-Foivos Thanos, Sophia Havaki, Dimitris Kletsas, Theocharis Panaretakis, Christopher J. Logothetis, Dimitris Stellas, Russell Petty, Giovanni Blandino, Angelos Papaspyropoulos, Vassilis G. Gorgoulis

**Affiliations:** 1 Department of Histology and Embryology, Molecular Carcinogenesis Group, Medical School, National and Kapodistrian University of Athens, Athens, Greece; 2 Biomedical Research Foundation, Academy of Athens, Athens, Greece; 3 Department of Basic Medical Sciences, Laboratory of Biology, Medical School, National and Kapodistrian University of Athens, Athens, Greece; 4 Department of Genitourinary Medical Oncology, The University of Texas MD Anderson Cancer Center, Houston, TX 77030 USA; 5 Institute of Chemical Biology, National Hellenic Research Foundation, 11635 Athens, Greece; 6 Laboratory of Cell Proliferation and Ageing, Institute of Biosciences and Applications, National Centre for Scientific Research “Demokritos”, Aghia Paraskevi, Greece; 7 Ninewells Hospital and Medical School, University of Dundee, Dundee, UK; 8 Department of Research, Oncogenomic and Epigenetic Unit, Diagnosis and Innovative Technologies, IRCCS Regina Elena National Cancer Institute, Rome, Italy; 9 Faculty Institute for Cancer Sciences, Manchester Academic Health Sciences Centre, University of Manchester, Manchester, UK; 10 Center for New Biotechnologies and Precision Medicine, Medical School, National and Kapodistrian University of Athens, Athens, Greece; 11 Faculty of Health and Medical Sciences, University of Surrey, Guildford, UK

**Keywords:** Prostate cancer, AR signaling blockade, Enzalutamide, GATA2-CDC6 axis, Cellular senescence

## Abstract

**Background:**

Prostate cancer is a major cause of cancer morbidity and mortality in men worldwide. Androgen deprivation therapy (ADT) has proven effective in early-stage androgen-sensitive disease, but prostate cancer gradually develops into an androgen-resistant metastatic state in the vast majority of patients. According to our oncogene-induced model for cancer development, senescence is a major tumor progression barrier. However, whether senescence is implicated in the progression of early-stage androgen-sensitive to highly aggressive castration-resistant prostate cancer (CRPC) remains poorly addressed.

**Methods:**

Androgen-dependent (LNCaP) and –independent (C4-2B and PC-3) cells were treated or not with enzalutamide, an Androgen Receptor (AR) inhibitor. RNA sequencing and pathway analyses were carried out in LNCaP cells to identify potential senescence regulators upon treatment. Assessment of the invasive potential of cells and senescence status following enzalutamide treatment and/or RNAi-mediated silencing of selected targets was performed in all cell lines, complemented by bioinformatics analyses on a wide range of in vitro and in vivo datasets. Key observations were validated in LNCaP and C4-2B mouse xenografts. Senescence induction was assessed by state-of-the-art GL13 staining by immunocytochemistry and confocal microscopy.

**Results:**

We demonstrate that enzalutamide treatment induces senescence in androgen-sensitive cells via reduction of the replication licensing factor CDC6. Mechanistically, we show that CDC6 downregulation is mediated through endogenous activation of the GATA2 transcription factor functioning as a *CDC6* repressor. Intriguingly, GATA2 levels decrease in enzalutamide-resistant cells, leading to CDC6 stabilization accompanied by activation of Epithelial-To-Mesenchymal Transition (EMT) markers and absence of senescence. We show that CDC6 loss is sufficient to reverse oncogenic features and induce senescence regardless of treatment responsiveness, thereby identifying CDC6 as a critical determinant of prostate cancer progression.

**Conclusions:**

We identify a key GATA2-CDC6 signaling axis which is reciprocally regulated in enzalutamide-sensitive and -resistant prostate cancer environments. Upon acquired resistance, GATA2 repression leads to CDC6 stabilization, with detrimental effects in disease progression through exacerbation of EMT and abrogation of senescence. However, bypassing the GATA2-CDC6 axis by direct inhibition of CDC6 reverses oncogenic features and establishes senescence, thereby offering a therapeutic window even after acquiring resistance to therapy.

**Supplementary Information:**

The online version contains supplementary material available at 10.1186/s13046-023-02769-z.

## Introduction

Prostate cancer is the second leading cause of cancer-related death in men in the USA, and a major cause of cancer morbidity and mortality worldwide, resulting in around 34,500 deaths in 2022 in the USA [1]. At an early stage, almost all types of prostate cancer are dependent on the presence of androgens activating the androgen receptor (AR) signaling pathway, thereby fostering cell survival [2–5]. Consequently, androgen deprivation therapy (ADT) is implemented for early-stage disease management as it reduces testosterone levels produced by the testicles, achieved either by surgical or medical castration [6–8]. Although ADT has proven effective for early-stage hormone-sensitive disease, prostate cancer eventually progresses to an androgen-independent state leading to metastatic events in the majority of patients [9, 10]. The mechanisms underlying acquired resistance to therapy still remain elusive.

Radiation therapy and a variety of anti-cancer agents have been shown to trigger various cellular responses, including the induction of senescence in cancer cells [11]. Cellular senescence is a generally irreversible cellular state characterized by permanent cell cycle arrest, justifying its role as an anti-tumor barrier [12]. Senescent cells remain metabolically active and may affect neighboring cells through secretion of a broad spectrum of factors, which are collectively referred to as senescence associated secretory phenotype (SASP) [12]. ADT was found to induce senescence in prostate cancer cells in vitro and in vivo [13, 14], however the effects were not permanent as cells ultimately progressed to the CRPC type [13, 15]. The molecular determinants of that transformation and how exactly senescence may be implicated in the process remain unclear.

Interestingly, the cardinal replication licensing machinery factor Cell Division Cycle 6 (CDC6) comprises an AR transcriptional target [16], however the role of CDC6 in prostate cancer has not been thoroughly addressed. Aberrant CDC6 overexpression leads to replication stress, thus fueling genomic instability and fostering malignant behavior [17–19]. Additionally, CDC6 exerts its oncogenic activity by functioning as a transcriptional repressor of the epithelial marker E-cadherin and inducing Epithelial-To-Mesenchymal Transition (EMT) [18, 20]. According to our oncogene-induced DNA damage model for cancer development, while CDC6 upregulation leads to replication stress-mediated senescence [21, 22], CDC6 constitutive activation eventually drives “escape” from senescence, whereby a proportion of senescent cells become capable of re-initiating proliferation through distinct genetic and epigenetic events, a direct consequence of accumulated genomic instability [23–25]. However, it remains unknown whether CDC6 may modulate senescence in the prostate cancer context with clinically meaningful outcomes.

Here, we implement the nonsteroidal AR inhibitor enzalutamide to elucidate molecular determinants governing prostate cancer progression from treatment-responsive to treatment-resistant forms. We confirm that AR blockade differentially impacts the two prostate cancer types, and identify CDC6 as a critical modulator of response to therapy. Mechanistically, we show that enzalutamide treatment significantly limits the invasive potential of androgen-sensitive prostate cancer cells by eliciting stringent upstream regulation of CDC6, resulting in senescence induction. In contrast, enzalutamide may further exacerbate the invasive potential of therapy-resistant CRPC cells and completely abrogate senescence. Importantly, we demonstrate that modulation of CDC6 signaling may reverse oncogenic phenotypes and establish senescence even in enzalutamide-resistant CRPC cells.

## Methods

### Cell lines

The human prostate cancer cell lines LNCaP and enzalutamide-resistant C4-2B were kindly provided by MDAnderson Cancer Center in Houston, Texas of USA. The enzalutamide-resistant PC-3 cell line was purchased by ATCC. All cell lines were cultured in RPMI medium supplemented with 10% fetal bovine serum (FBS) and 1% penicillin/streptomycin, were maintained at 37 °C and 5% CO_2_ and grown to 70% confluency.

### AR signaling inhibition

The pharmacological blockade of AR signaling pathway in all cell lines was achieved after 5-day treatments with Enzalutamide at a final concentration of 10 μΜ (stock concentration 10 mM). Enzalutamide was replenished in the cell culture medium every 2 days. As control, DMSO was added in the cell culture medium at 0.1% final concentration. Enzalutamide (MDV3100) was purchased from Selleckchem (Catalog No. S1250).

### RNA interference (RNAi) experiments

For siRNA-mediated silencing in LNCaP, C4-2B and PC-3 cell lines, Lipofectamine 2000 (Invitrogen) was used according to manufacturer instructions. The following oligonucleotides were used for siRNA-mediated silencing: non-targeting (CTRL): UAAGGUAUGAAGAGAUAC (Dharmacon), siRNAs to GATA2 (set of 4, GS2624) were purchased from Qiagen (stock concentration 10 μM, used concentration 100 nM) and CDC6 Stealth siRNAs (set of 3; HSS101647, HSS101648, HSS101649) were purchased from ThermoFischer Scientific (1299001) (stock concentration 20 μM, used concentration 150 nM).

### Protein extraction and Western Blot analysis

For total protein extraction, cells and tissue samples were washed with cold PBS prior to lysis with Laemmli Lysis Buffer (50 mM Tris–HCl pH 6.8, 2% SDS, 50 mM NaF, 10 mM β-glycerophosphate, 0.5 mM Na_3_VO_4_ and 1 × EDTA free protease inhibitors (Roche)) and then the cell lysates were normalized by NanoDrop (Thermo Scientific). Western blotting was carried out as previously described [26]. Briefly, samples were loaded on 8–10% acrylamide/bis-acrylamide gels and then transferred onto PVDF membrane (Millipore) before immunoblotting with the appropriate primary antibodies overnight at 4 °C. Primary antibodies were used at the following concentrations; AR (dilution 1:000, Cell Signaling #5153), PSA (dilution 1:1000, Cell Signaling #2475), CDC6 (dilution 1:250, Santa Cruz #9964), p21^WAF1/Cip1^ (dilution 1:500, Cell Signaling #2947), E-Cadherin (CDH1; dilution 1:500, Cell Signaling #3195) and GAPDH (dilution 1:2000, Cell Signaling #5174). Anti-mouse (Cell Signaling #7076) and anti-rabbit (Cell Signaling #7074) HRP-linked secondary antibodies were used at a dilution of 1:1000.

### Cell proliferation assay

Potential proliferation effects after treatment with Enzalutamide were determined by 3-(4,5-dimethylthiazol-2-yl)-2,5-diphenyltetrazolium bromide (MTT) assays (CellTiter 96® Non-radioactive Cell Proliferation Assay, Promega) in LNCaP and C4-2B cells. For the experiments, 8000 cells from each cell line were seeded into a 96-well plate in octuplicate, and were incubated in a 37 °C, 5% CO_2_ incubator_._ Cells were treated either with Enzalutamide or DMSO as control. All samples were processed according to the manufacturer’s instructions. After adding the MTT solution in the culture medium, cells were incubated for 2–4 h. Absorbance was then measured at 570 nm using a 96-well plate reader (Anthos 2010 Microplate Reader, Biochrom). Three control wells with only culture medium were used to obtain the blanks of absorbance.

### In vitro migration and invasion assays

PC-3 cells were cultured in 6-well dishes, in DMEM containing 10% FCS. Once at confluence, the medium was changed with fresh one containing or not enzalutamide at a final concentration of 10 μM. To assess migration, three days later the cell layer was scraped in a straight line using a 200 μl pipette tip. Floating cells were removed and the remaining cells were washed with PBS and fresh medium (with or without enzalutamide) was again added. The cultures were photographed under a Nikon Eclipse TS2 microscope immediately after scratching (0 h) and after 24 h. The invasive potential of enzalutamide-treated and -untreated PC-3 cells was estimated using a cell invasion assay kit (Chemicon), according to the manufacturer’s instructions.

### RNA extraction, cDNA preparation and qRT-PCR

RNA from cells and tissue samples was isolated using Nucleospin RNA (Macherey–Nagel #740,955) based on the manufacturer’s instructions. cDNA synthesis was carried out using the GoScript Reverse Transcriptase kit (Promega; A5000). Quantitative Real-Time PCR was performed as previously described [26, 27] following the Power SYBR Green Cells-to-Ct kit protocol (Applied Biosystems). After cDNA preparation, the cDNA was diluted down 4 times in Nuclease-free water. The PCR Cocktail mix was then produced including the forward and reverse primers. PCR reactions were carried out in 96-well plates (Life Technologies) and the PCR instrument (Applied Biosystems) was programmed as follows: Stage 1; Enzyme activation (hold), repeats 1, temperature 95 °C, time 10 min, Stage 2; PCR (cycle), repeats 40, Step 1; Temperature 95 °C, time 15 s, Step 2; Temperature 60 °C, time 1 min, Stage 3. Dissociation curve Step 1; Temperature 95 °C, time 15 s. Step 2; Temperature 60 °C, time 1 min, Step 3; Temperature 95 °C, time 30 s, Step 4; Temperature 60 °C, time 15 s. The primer sequences that were used are the following: E-CADHERIN (CDH1) forward: 5’-GCCTCCTGAAAAGAGAGTGGAAG-3’ and reverse 5’-TGGCAGTGTCTCTCCAAATCCG-3’, SNAIL (SNAI1) forward 5’- TGCCCTCAAGATGCACATCCGA-3’ and reverse 5’- GGGACAGGAGAAGGGCTTCTC-3’, ZEB1 forward 5’- GGCATACACCTACTCAACTACGG-3’ and reverse 5’- TGGGCGGTGTAGAATCAGAGTC-3’, GATA2 forward 5’- CAGCAAGGCTCGTTCCTGTTCA-3’ and reverse 5’- ATGAGTGGTCGGTTCTGCCCAT-3’, GAPDH forward 5’- TGCACCACCAACTGCTTAGC-3’ and reverse 5’- GGCATGGACTGTGGTCATGAG-3’. The results were averaged from three independent experiments and further analysis was conducted using the 2^-ΔΔCt method.

### Immunofluorescence

Cells were seeded on coverslips (70% confluency), fixed (4% PFA/PBS, 10 min in 4 °C) and permeabilized applying Triton-X 0.3%/PBS for 15 min. Blocking of non-specific binding of antibodies was performed by applying normal goat serum for 1 h at RT (dilution 1:40, Abcam ab138478). Then, cells were stained for lipofuscin detection using SenTraGor™ reagent as described in the SenTraGor™ staining section below. To visualize senescent cells, primary anti-biotin conjugated fluorescent antibody (dilution 1:100, Biotium BNC610400-100) was used for 1 h at RT. Cells were washed and stained with primary antibody anti-p21^WAF1/Cip1^ (dilution 1:200, Cell Signaling #2947) for 1 h at RT. After washing with PBS, secondary antibody (Goat Anti-Rabbit: dilution 1:500, Abcam ab150077) was applied for 1 h at RT. Cells were washed with PBS and cell nuclei were counterstained with DAPI. Mounting of cells was carried out after washing with dH_2_0 for 30 s. Cells were observed using the Leica TCS-SP8 confocal microscope on the 10 × Objective (100 × magnification).

### Immunocytochemistry

Cells were seeded, fixed, permeabilized and blocked as described in the Immunofluorescence section. SenTraGor™ reagent was applied as described in the SenTraGor™ staining section below. Primary anti-biotin antibody (dilution 1:300, Abcam ab201341), an anti-Ki67 (dilution 1:200, Abcam ab16667) and an anti-cleaved Caspase-3 (dilution 1:300, Cell Signaling #9664) antibody were applied for 1 h at RT. Development of positive signal was performed using the Dako REAL EnVision Detection System, (Cat.no: K5007) according to the manufacturer’s instructions. Cells were stained with Hematoxylin and mounted. Cells were observed using a ZEISS Axiolab5 microscope on the 10x, 20 × or 40 × Objectives (100x, 200 × and 400 × magnification, respectively).

### Immunohistochemistry

4 μm sections from formalin-fixed paraffin embedded (FFPE) mouse tumors were obtained. Tumor sections were deparaffinized, hydrated and antigen retrieval was performed by heating them in 10 mM citric acid (pH 6.0) for 10 min. SenTraGor™ reagent was applied as described in the SenTraGor™ staining section below. Blocking of non-specific binding of antibodies was performed by applying normal goat serum for 1 h at RT (dilution 1:40, Abcam ab138478). Then primary anti-biotin antibody (dilution 1:300, Abcam ab201341) and primary anti-Ki67 (dilution 1:200, Abcam ab16667) were applied and the sections were incubated overnight at 4 ^◦^C. Development of positive signal was performed using the Dako REAL EnVision Detection System, (Cat.no: K5007) according to the manufacturer’s instructions. Tumor sections were observed using a ZEISS Axiolab5 microscope on a 20 × Objective (200 × magnification).

### SenTraGor™ (GL13) staining for senescence detection

SenTraGor™ staining was carried out as previously described [28, 29]. More specifically, cells seeded on coverslips and tissue sections after blocking were treated in the beginning with 50% ethanol for 5 min and then with 70% ethanol for 5 min. SenTraGor™ was applied for 10 min at 37 °C. Coverslips/tissue sections were washed with 50% ethanol for 2 min and then with 1X PBS. Excess amount of SenTraGor™ was removed by a 3-min wash with Triton-X 0.3%/PBS. Cells/tissue sections were washed with PBS and primary anti- biotin antibodies were applied for 1 h at RT (dilution 1:300, Abcam ab201341 in the case of immunocytochemistry and immunohistochemistry; dilution 1:100, Biotium BNC610400-100 in the case of immunofluorescence).

### SA-β-galactosidase cell staining for senescence detection

LNCaP, C4-2B and PC-3 cells were seeded and cultured in 6-well plates. After 5 days of treatment, cells were washed once with 1X PBS and fixed (Cell Signaling #9860) for 10 min at RT. Cells were then washed three times with 1X PBS and incubated with 1 ml of β-galactosidase staining solution (Cell Signaling #9860) overnight at 37 °C in a dry incubator (no CO_2_). The plates were sealed with parafilm to prevent evaporation. After the overnight incubation, cells were observed using the Leica DFC-320 phase contrast microscope on a 10 × Objective (100 × magnification).

### Scanning electron microscopy

LNCaP and C4-2B cells were cultured up to 80% confluence and fixed in 2.5% glutaraldehyde in 0.01 M PBS for 30 min at room temperature (RT). They were then harvested using a scraper, collected into a tube and centrifuged at 800 × g for 5 min at RT. The supernatant was aspirated, while cells were resuspended in warmed 4% gelatin aquatic solution followed by centrifugation at 800 × g for 5 min at RT and cooled on ice. Under a stereoscope the solidified cell pellet with gelatin was extracted, cut into small fragments (1–2 mm^3^) and transferred into PBS at 4 °C. The cell-gelatin fragments were then dehydrated in graded series of ethyl alcohol, followed by propylene oxide (PO) treatment, infiltrated gradually in a mixture of Epon/Araldite resins diluted in PO, and finally embedded in fresh epoxy resin mixture. Ultrathin sections (70–90 nm thickness) were cut on a Leica Ultracut R ultramicrotome, equipped with a Diatome diamond knife, and mounted onto 200-mesh copper grids. The sections were then counterstained with ethanolic uranyl acetate followed by lead citrate and observed on a Philips 420 transmission electron microscope equipped with an Olympus Megaview G2 CCD camera.

### In vivo experiments

All animal studies were approved by the National Hellenic Research Foundation (NHRF) Animal Care and Use Committee. The study protocol was approved by the local ethics committee (Athens Prefecture Veterinarian Service; (315856/15–03-2023)). The in vivo study was conducted in the ISO 9001: 2015 operating (registration number I-030–02-100–01430) animal model unit of the Institute of Chemical Biology of the NHRF. Briefly, male C.B-Igh-1^b^/IcrTac-Prkdc^scid^ (SCID) mice (*n* = 6 per condition) were subcutaneously inoculated with 5 × 10^6^ LNCaP or C4-2B cells resuspended in BME (R&D Systems). When the tumors became palpable (around 50 mm^3^ in size), the mice were treated with 10 mg/kg enzalutamide diluted in DMSO and resuspended in corn oil/saline solution (the final DMSO concentration was 0.001% w/v). Enzalutamide was administrated by oral gavage for 8 consecutive days. The mice were monitored on a daily basis for any signs of illness or discomfort. At the end of the experiment, the mice were euthanized and the tumors were surgically excised for downstream molecular analysis and immunohistochemistry.

### RNA sequencing and bioinformatics analysis

Total RNA was extracted from LNCaP cells after 5 days of treatment with 10uM Enzalutamide or vehicle (DMSO) using the Nucleospin RNA (Macherey–Nagel #740955) kit. RNA samples were processed by the Sequencing and Microarray Facility (SMF) of the MD Anderson Cancer center (MDACC). Next generation sequencing was performed on a HiSeq4000 sequencer (Illumina). Briefly, RNA samples treated with DNase-1 were assessed for size distribution using the Fragment Analyzer High Sensitivity RNA Analysis Kit (Advanced Analytical) and by quantity with the Qubit RNA HS Assay Kit (Thermo Fisher Scientific). RNA was converted to double-stranded cDNA, then amplified using Nugen's proprietary single primer isothermal (Ribo-SPIA) protocol. The resulting cDNAs were fragmented to an average size of 200 bp, and libraries were constructed using the KAPA Hyper Library Preparation Kit, followed by two cycles of PCR library enrichment. Following cleanup, the libraries were mixed (three libraries per pool), then quantified by qPCR using the KAPA Library Quantification Kit (KAPA Biosystems) and sequenced with one pool per lane on the Illumina HiSeq4000 Sequencer using a 75 bp paired-end format. In order to compare the transcriptome of enzalutamide-treated LNCaP cells versus untreated control, normalization of reads and removal of unwanted variation was performed with RUVseq [30]. Differentially expressed genes were identified using the DESeq 2 [31]. R package and genes with log2 fold change cut-off of 1.5 and p-value less than 0.05 were considered to be significant. Gene ontology and pathway analysis was performed using the Database for Annotation, Visualization and Integrated Discovery (DAVID)[32]. Only pathways and biological processes with p-value equal or less than 0.05 were considered to be significantly enriched.

Publically available RNA-seq raw data for enzalutamide-sensitive and enzalutamide- resistant human samples with accession numbers GSE55030 [33], GSE109708 [34], GSE184168 [35], GSE163240 [36], GSE150807 [37] and GSE151083 [38] were downloaded from ENA UK browser (https://www.ebi.ac.uk/ena/browser/home). Sequence adapters were trimmed (if needed) using TrimGalore (https://www.bioinformatics.babraham.ac.uk/projects/trim_galore/) and then reads were mapped to human genome version GRCh38/hg38 using STAR [39] aligner. SAMtools [40] was used for data filtering and file format conversion, while the HT-seq count algorithm [41] was used to assign aligned reads to exons using the following command line “htseq-count –s no –m intersection –nonempty”. Normalization of reads and removal of unwanted variation was performed with RUVseq [30]. Differentially expressed genes were identified using the DESeq2 R package [31] and genes with log2 fold change cut-off of 0.5 and adjusted p-value less than 0.05 were considered to be significant. Gene ontology and pathway analysis was performed as described above.

### Statistical analysis

All observations reported in this manuscript were based on at least three biological replicates, and statistical significance was derived using Student’s t-test.

## Results

### A GATA2-CDC6 axis regulates senescence in response to AR blockade

**Fig. 1:**
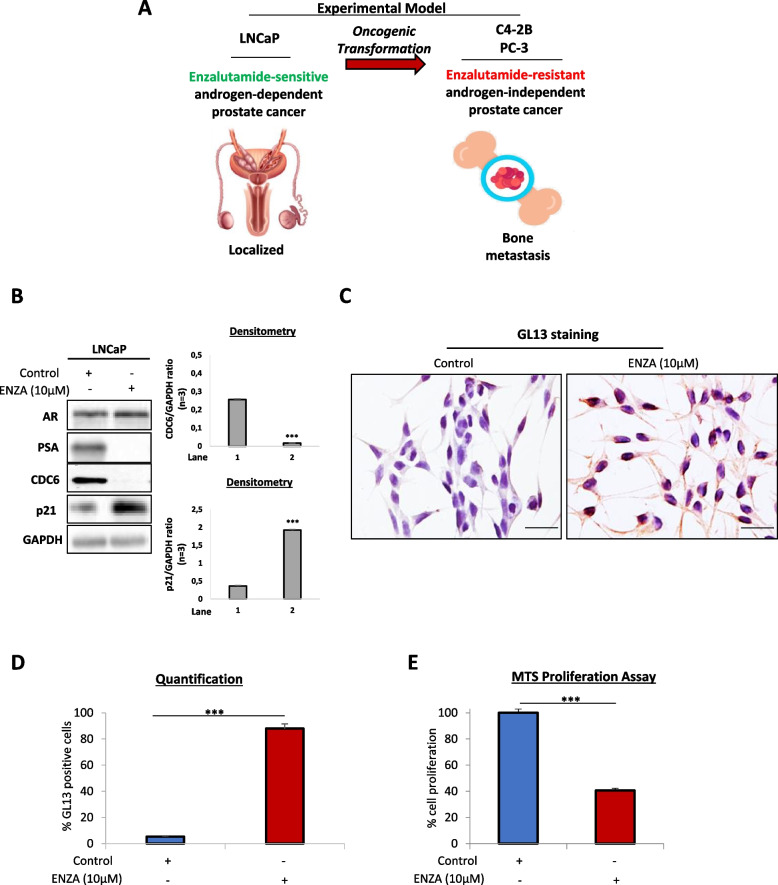
Inhibition of AR signaling limits CDC6 and induces senescence in androgen-sensitive prostate cells. **A** Schematic demonstrating prostate cancer development. The androgen-dependent LNCaP prostate cancer cell line represents enzalutamide-sensitive localized cancer. LNCaP-derived C4-2B prostate cancer cells are isolated from bone metastases and constitute androgen-resistant cells. PC-3 prostate cancer cells are also isolated from metastatic to bone adenocarcinoma and represent androgen-resistant cells. **B** Western blotting and densitometry of control and enzalutamide (ENZA)-treated LNCaP cell lysates with the indicated antibodies. Complete absence of PSA expression upon enzalutamide treatment verifies responsiveness to treatment. GAPDH was used as endogenous control. **C** GL13 immunocytochemical staining of control and enzalutamide-treated LNCaP cells. Lipofuscin predominantly accumulates in the cytoplasm of treated LNCaP cells. Magnification: 200x (Objective 20x), scale bars: 30 μm. See also Fig. S1A and B. **D** Quantification of C. **E** MTS proliferation assay verifying reduced proliferation of enzalutamide-treated LNCaP cells. Enzalutamide was used at a 10 μM concentration. ***P < *0.001* of Student’s t-test. Error bars indicate s.e.m. Data shown are representative of at least three biological replicates (n ≥ 3). Vectors were obtained from www.vecteezy.com

To investigate the molecular basis of sensitivity to AR blockade, we initially implemented the LNCaP prostate cancer cell line, representing androgen-dependent localized tumors (Fig. 1A). AR was previously found to directly induce CDC6 transcription [16]. Moreover, androgen deprivation was shown to induce senescence in LNCaP cells, however with no permanent effects, ultimately leading to CRPC development [13–15]. To that end, we initially sought to determine whether AR blockade may induce senescence involving CDC6.

We found that treatment of LNCaP cells with the AR inhibitor enzalutamide completely abrogated CDC6 expression, in line with the previously reported role of AR in CDC6 transcription (Fig. 1B). Interestingly, although senescence as a stress response mechanism was previously shown to be triggered by CDC6 upregulation [21], CDC6 downregulation in LNCaP cells was accompanied by p21^WAF1/Cip1^ increase, a p53 downstream target involved in senescence induction [12, 42] (Fig. 1B). Establishment of senescence upon enzalutamide treatment was further validated by immunocytochemical GL13 staining identifying lipofuscin accumulation, one of the most prominent senescence hallmarks [29], as well as SA-β-gal staining (Fig. 1C-D and S1A-B). In line with the senescent phenotype, cellular proliferation was decreased in cells receiving treatment (Fig. 1E).

**Fig. 2:**
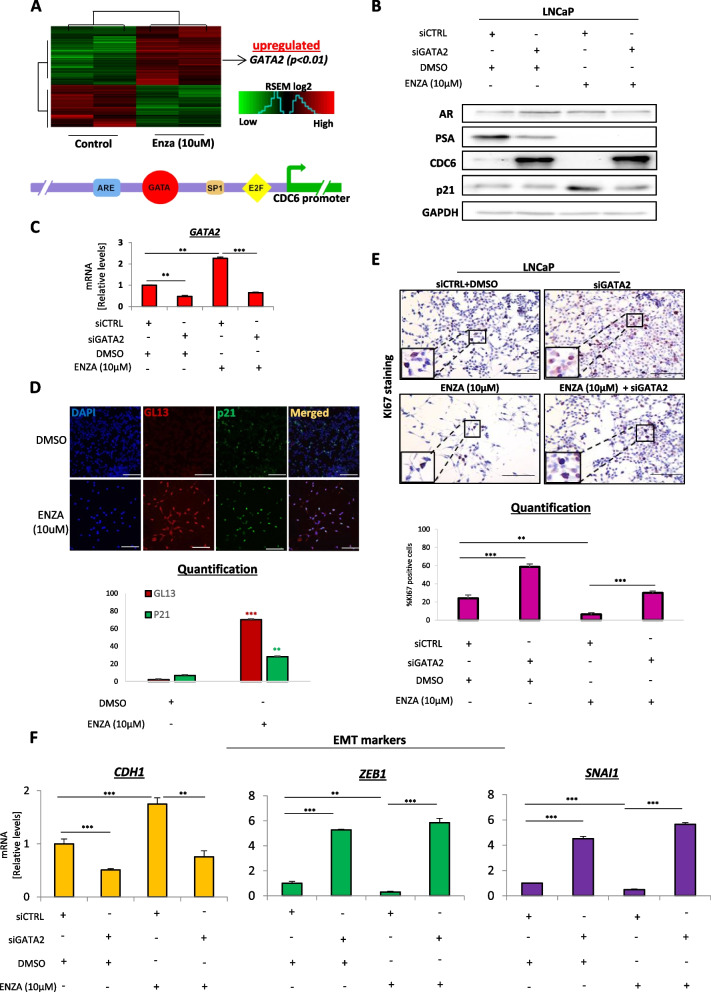
Enzalutamide elicits GATA2-mediated CDC6 suppression in responsive cells.** A** RNA sequencing analysis in control and enzalutamide-treated LNCaP cells identified GATA2 as one of the most significantly upregulated factors (*P* < *0.01*). See also Table S1. GATA elements have been identified in the *CDC6* promoter, adjacent to putative Androgen Response Element (ARE) sites [16]. **B** Western blotting in LNCaP cells transfected with the indicated siRNAs and/or treated with DMSO or enzalutamide. siCTRL indicates non-targeting siRNA. Cell lysates were probed with the indicated antibodies. CDC6 is upregulated upon GATA2 depletion regardless of treatment. See also Fig. S2A. **C** qPCR for GATA2 mRNA levels validating successful GATA2 KD in B. Endogenous GATA2 levels are upregulated in response to enzalutamide. **D** Immunofluorescence for dual GL13/p21.^WAF1/Cip1^ staining (top) and quantification (bottom) confirms senescence induction only in enzalutamide-treated cells in B. **E** Ki67 immunocytochemical staining (top) and quantification (bottom) of cells in B indicate proliferation levels proportional to CDC6 expression. Magnification: 100x (Objective 10x), scale bars: 60 μm. Inset magnification: 400x (Objective 40x). **F** qPCR for EMT markers *CDH1*, *ZEB1* and *SNAI1* in the indicated conditions of cells from B. GATA2 depletion is accompanied with an EMT marker expression profile regardless of treatment. Data are normalized to GAPDH expression. Enzalutamide was used at a 10 μΜ concentration. **P < *0.01* and ***P < *0.001*, of Student’s t-test; n.s., non-significant. Error bars indicate s.e.m. Data shown are representative of at least 3 biological experiments (n ≥ 3)

To elucidate the mechanism underlying induction of senescence upon CDC6 reduction, we carried out RNA sequencing in control and enzalutamide-treated LNCaP cells with the scope of uncovering important regulators of the process. Interestingly, we identified the transcription factor GATA2 as one of the most significantly upregulated targets in response to therapy (Fig. 2A and Table S1). Notably, GATA elements have been previously detected in the *CDC6* promoter of LNCaP cells, in the vicinity of putative Androgen Response Element (ARE) sites [16] (Fig. 2A), implying a potentially direct role of GATA factors in CDC6 transcription regulation. To investigate a possible GATA2-mediated CDC6 regulation, we silenced GATA2 in LNCaP control or enzalutamide-treated cells, and observed CDC6 stabilization in response to GATA2 loss regardless of treatment (Fig. 2B, C and S2A). Of note, GATA2 silencing failed to induce p21^WAF1/Cip1^ expression even upon treatment (Fig. 2B, C and S2A). Intriguingly, endogenous GATA2 levels were found to be significantly upregulated in response to enzalutamide, accompanied by CDC6 loss, p21^WAF1/Cip1^ increase and induction of senescence (Fig. 2B-D). Those results demonstrate that AR blockade in androgen-sensitive LNCaP cells leads to natural activation of GATA2, which may act as a direct repressor of CDC6, thereby allowing establishment of senescence.

We next investigated the impact of the potential GATA2-CDC6 axis on LNCaP oncogenic behavior. As expected, and reciprocally to the acquisition of senescent features, cellular proliferation was found significantly increased upon siGATA2-mediated CDC6 stabilization, assessed by Ki67 expression (Fig. 2E). Moreover, loss of GATA2 led to activation of EMT, as shown by reduced levels of E-cadherin (CDH1) and increased expression of ZEB1 and SNAI1 (Fig. 2F). Those results indicate that upon GATA2 loss, CDC6 upregulation may be sufficient to confer invasive features to localized prostate cancer cells, thus contributing to their metastatic transformation.

### Loss of CDC6 establishes senescence in enzalutamide-resistant prostate cancer cells

We next sought to explore whether AR blockade in a CRPC setting may again rely on modulation of CDC6 expression, as demonstrated in androgen-sensitive cells. For that purpose, we made use of androgen-independent C4-2B and PC-3 cells. C4-2B cells are isolated from bone metastases formed in nude mice following inoculation with LNCaP-derived, androgen-independent C4-2 cells [43]. As C4-2B cells are enzalutamide-resistant, together with LNCaP, they constitute an excellent preclinical model to investigate progression of localized androgen-sensitive into metastatic androgen-independent disease (Fig. 1A) [44]. PC-3 cells are derived from metastatic to bone human prostate adenocarcinoma and are also shown to exhibit increased resistance to enzalutamide (Fig. 1A) [45, 46].

**Fig. 3:**
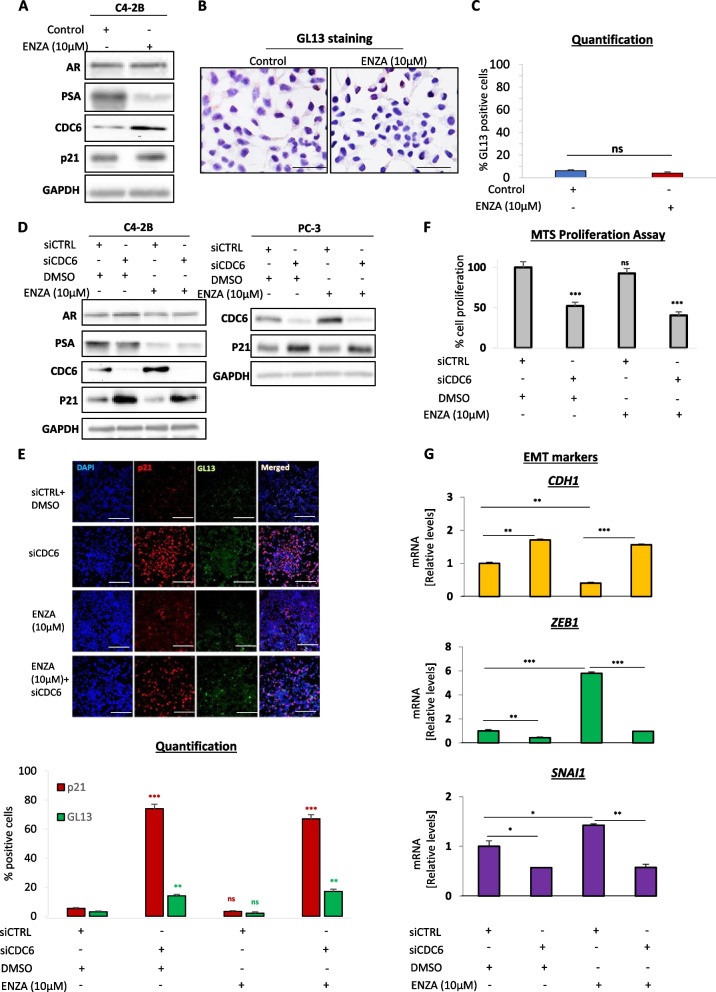
Loss of CDC6 rescues the oncogenic potential of ezalutamide-resistant cells and establishes senescence.** A** Western blotting in C4-2B cells receiving or not additional treatment with enzalutamide. Cell extracts were blotted with the indicated antibodies. CDC6 is stabilized upon treatment. See also Fig. S2B. **B** GL13 immunocytochemical staining of C4-2B cells to assess senescence induction upon enzalutamide treatment. Magnification: 200x (Objective 20x), scale bars: 30 μm. See also Fig. S1C and D. **C** Quantification of cells in B displaying non-significant differences between the indicated conditions. **D** Western blotting in C4-2B and PC-3 cells transfected with the indicated siRNAs and/or treated with DMSO or enzalutamide. Cell lysates were probed with the indicated antibodies. CDC6 is stabilized upon enzalutamide treatment at the expense of p21^WAF1/Cip1^. See also Fig. S2C and D. **E** Immunofluorescence for dual GL13/p21^WAF1/Cip1^ staining (top) and quantification (bottom) confirms senescence induction only in CDC6-depleted cells in D. Magnification: 100x (Objective 10x), scale bars: 60 μm. See also Fig. S1E-F and S3. **F** MTS proliferation assay in C4-2B cells from D displaying a proliferation decrease following CDC6 depletion. See also Fig. S4A-D. **G** qPCR for EMT markers *CDH1*, *ZEB1* and *SNAI1* in the indicated conditions of cells from D. CDC6 depletion rescues EMT marker expression, while enzalutamide-mediated CDC6 stabilization has the opposite effect. Data are normalized to GAPDH expression. Enzalutamide was used at a 10 μΜ concentration. *P < *0.05*, **P < *0.01* and ***P < *0.001*, of Student’s t-test; n.s., non-significant. Error bars indicate s.e.m. Data shown are representative of at least 3 biological experiments (n ≥ 3)

Interestingly, treatment of C4-2B with enzalutamide resulted in an increase of CDC6 levels, while no senescence induction was observed as indicated by unaltered p21^WAF1/Cip1^ levels and lack of GL13/SA-β-gal staining (Fig. 3A-C, S1C-D and S2B). This observation urged us to test the possibility that loss of CDC6 may reverse the C4-2B cell phenotype. To exclude the possibility of cell line bias, we independently validated those findings additionally in PC-3 cells. Indeed, knockdown of CDC6 in C4-2B and PC-3 cells was sufficient to lead to upregulation of p21^WAF1/Cip1^ levels and induction of senescence (Fig. 3D-E, S1E-F, S2C-D and S3). Importantly, CDC6 silencing led to senescence even after enzalutamide treatment (Fig. 3D-E, S1E-F, S2C-D and S3).

In accordance with the senescent phenotype induced by CDC6 loss, CDC6-depleted cells exhibited significantly reduced proliferation (Fig. 3F and S4A-D). Along the same lines, CDC6 depletion limited EMT, as indicated by the significantly altered expression of all tested EMT markers (Fig. 3G and S4E). Of note, enzalutamide treatment alone led to a marked activation of EMT (Fig. 3G and S4E), implying that CDC6 upregulation upon acquired resistance may actually exacerbate the invasive potential of prostate cancer cells. Taken together, our results indicate a potentially opposite regulation pattern of CDC6 in androgen-dependent and –independent prostate cancer cells; however, suppression of CDC6 may elicit senescence and rescue key oncogenic features of androgen-independent cancer cells.

### Enzalutamide regulates GATA2-CDC6 signaling to exacerbate EMT upon acquiring resistance to therapy

**Fig. 4:**
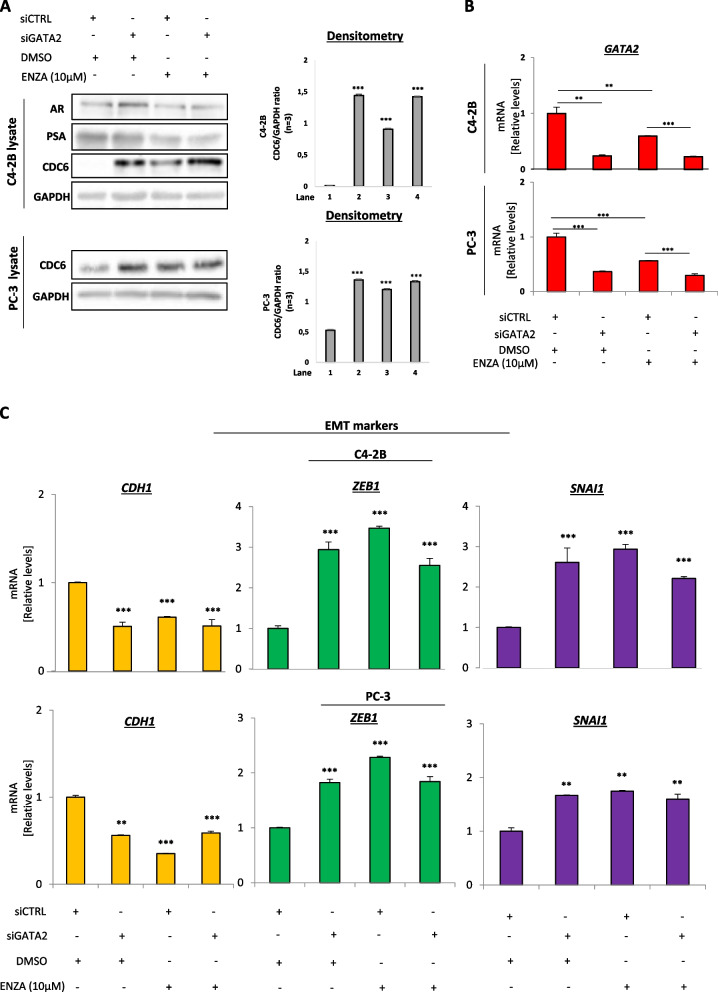
Enzalutamide induces GATA2-dependent CDC6 upregulation and further promotes EMT upon acquiring therapy resistance. **A** Western blotting and densitometry in C4-2B and PC-3 cells transfected with the indicated siRNAs and/or treated with DMSO or enzalutamide. Cell lysates were probed with the indicated antibodies. CDC6 is stabilized either upon GATA2 depletion or enzalutamide treatment. **B** qPCR for GATA2 mRNA levels validating successful GATA2 KD in A. Endogenous GATA2 levels are downregulated in response to therapy in already enzalutamide-resistant cells. See also Fig. S5. **C** qPCR for EMT markers *CDH1*, *ZEB1* and *SNAI1* in the indicated conditions of cells from A. GATA2 depletion or enzalutamide treatment induce EMT marker expression. Enzalutamide was used at a 10 μM concentration. **P < *0.01* and ***P < *0.001*, of Student’s t-test. Error bars indicate s.e.m. Data shown are representative of at least 3 biological experiments (n ≥ 3)

In order to investigate whether enzalutamide-mediated CDC6 upregulation may be again dependent on GATA2 in a castration-resistant environment, we silenced GATA2 in C4-2B and PC-3 cells receiving or not enzalutamide treatment. We found that in both cell lines GATA2 loss led to increased expression of CDC6 compared to control regardless of treatment (Fig. 4A and B), verifying again the function of GATA2 as a CDC6 suppressor. Interestingly, and in contrast to what we observed in LNCaP cells, enzalutamide treatment alone led to a significant reduction of endogenous GATA2 levels compared to control, which was accompanied by increased CDC6 protein levels (Fig. 4A and B, third lane).

To investigate the effects of GATA2-mediated CDC6 regulation in this setting, we again evaluated several oncogenic features of C4-2B and PC-3 cells transfected with siRNA against GATA2, in combination or not with enzalutamide treatment. We found no evidence of senescence following either GATA2 depletion or enzalutamide treatment (Fig. S5A-F), in line with CDC6 upregulation. Moreover, although we did not observe significant changes in proliferation capacity upon GATA2 depletion or drug treatment (Fig. S5G and H), there was a significant activation of EMT markers for both cell lines, highlighting that CDC6 stabilization through natural or RNAi-mediated GATA2 silencing is sufficient to confer more aggressive features to already metastatic prostate cancer cells (Fig. 4C). This was further confirmed via in vitro migration and invasion assays in PC-3 cells demonstrating acquisition of an enhanced migratory and invasive potential upon enzalutamide treatment (Fig. S6A-C). Our results demonstrate that the identified GATA2-CDC6 axis is differentially modulated in CRPC cells, where further treatment with AR inhibitors may actually lead to enhanced invasiveness through GATA2-dependent CDC6 upregulation. This is an important point to be considered in treating CRPC patients, as therapy-driven oncogenic effects may initiate immediately upon acquiring resistance.

**Fig. 5:**
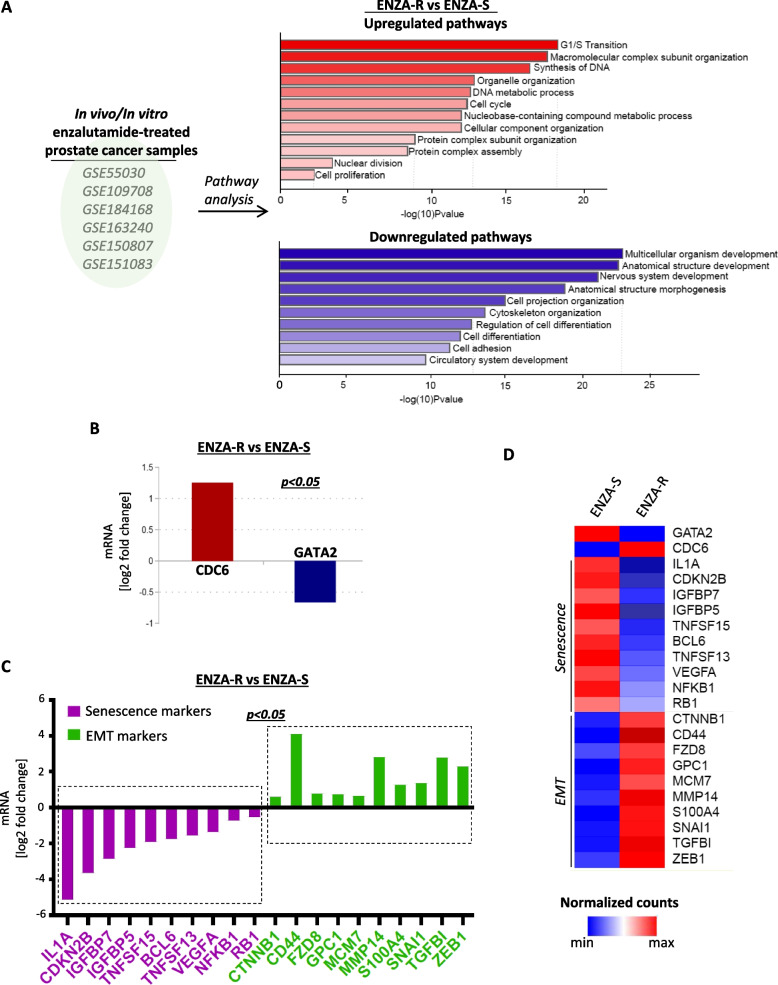
CDC6 and EMT inversely correlate with GATA2 and senescence in enzalutamide-sensitive and -resistant prostate cancer samples in vitro and in vivo. **A** Publically available RNA-seq datasets of enzalutamide-sensitive (ENZA-S) and –resistant (ENZA-R) prostate cancer cell lines or mouse xenografts were retrieved and grouped to conduct pathway enrichment analyses. Pathways related to cell cycle transition, cell division and proliferation were predominant in ENZA-R samples versus pathways related to cell adhesion. See also Table S2. **B** mRNA CDC6 levels are significantly higher in ENZA-R than ENZA-S samples, and the opposite applies for GATA2. **C** ENZA-R samples display significantly higher expression of known EMT markers [49], including ZEB1 and SNAI1, compared to ENZA-S counterparts. In contrast, ENZA-R samples display significantly lower expression of senescence and SASP markers compared to ENZA-S samples. Senescence and SASP markers were retrieved from the Reactome online tool (https://www.reactome.org/content/detail/R-HSA-2559582). **D** Normalized RNA seq count-based heatmap from all datasets demonstrating that increased CDC6 in ENZA-R samples directly correlates with elevated EMT markers, however it is reciprocal to GATA2 levels and senescence markers. The exact opposite pattern is observed in ENZA-S samples

In order to further validate our findings in a larger dataset, we retrieved publically available RNA-seq data from enzalutamide-resistant and -sensitive prostate cancer cell lines and tumor samples [33–38], and conducted a comparative pathway enrichment analysis between the two groups (Fig. 5A and Table S2). As expected, pathways related to cell cycle progression and active cell division were predominant in enzalutamide-resistant samples, whereas pathways linked to cell adhesion and differentiation were found downregulated (Fig. 5A). By further investigating the components of the predominant pathways we found that CDC6 was significantly elevated in enzalutamide-resistant samples compared to enzalutamide-sensitive counterparts, while GATA2 expression followed the opposite pattern (Fig. 5B), in keeping with our in vitro observations. We next sought to correlate the GATA2-CDC6 pattern with the presence of senescence and/or EMT across the whole dataset. Indeed, we found that a panel of well-characterized senescence markers [47, 48] were significantly downregulated in enzalutamide-resistant samples, while established EMT markers, including ZEB1 and SNAI1 [49], were found increased (Fig. 5C). A heatmap of normalized raw counts of all relevant genes clearly demonstrated the inverse correlation of CDC6 with GATA2 in both enzalutamide-resistant and –sensitive samples (Fig. 5D). Moreover, GATA2^low^ -CDC6^high^ levels correlated with EMT activation, whereas GATA2^high^-CDC6^low^ levels correlated with the presence of senescence signatures in all cases (Fig. 5D). Those results support our in vitro observations, where we demonstrate that experimental modulation of the GATA2-CDC6 axis may indeed be sufficient to rescue detrimental clinical phenotypes of AR blockade-resistant prostate cancer.

Although senescence detection was carried out using the established guidelines and multi-marker algorithm [29], we sought to safely exclude the possibility that the observed effects of GATA2-CDC6 modulation were not due to activation of other cellular processes, such as autophagy or apoptosis. To that end, we subjected both enzalutamide-treated and control LNCaP and C4-2B cells to scanning electron microscopy (SEM), and ruled out the possibility of autophagy induction, as no autophagic vacuoles were found in any condition (Fig. S7A). Moreover, LNCaP, C4-2B and PC-3 cells were all found negative for cleaved caspase-3 staining, irrespective of treatment (Fig. S7B and C), which additionally excluded induction of apoptosis as a potential outcome of enzalutamide treatment.

**Fig. 6:**
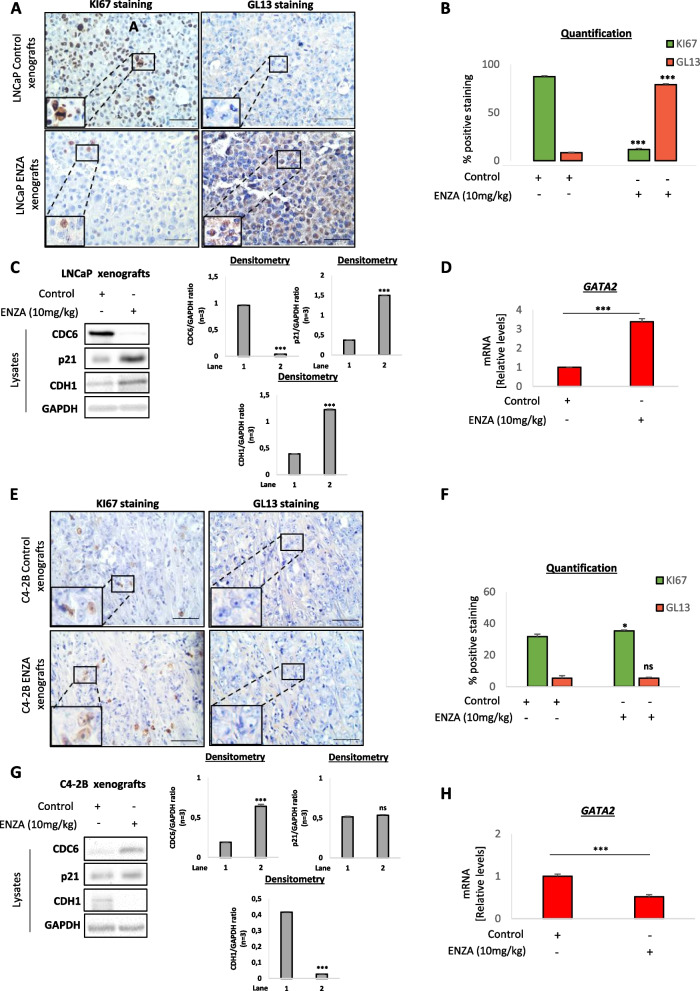
Effects of enzalutamide treatment are recapitulated in responsive and non-responsive prostate cancer xenografts.** A** Immunohistochemistry displaying Ki67 and GL13 stainings in serial sections of LNCaP mouse xenografts receiving enzalutamide (10 mg/kg) or no treatment (Control). Enzalutamide-treated xenografts display reduced proliferation and increased senescence compared to control. **B** Quantification of Ki67 and GL13 stainings in A. **C** Western blotting of cell lysates from LNCaP xenografts in A, using the indicated markers. Densitometry was carried out for all markers. See also Fig. S8A. **D** q-PCR for GATA2 levels in cell lysates from LNCaP xenografts in A. **E** Immunohistochemistry displaying Ki67 and GL13 stainings in serial sections of C4-2B mouse xenografts receiving enzalutamide (10 mg/kg) or no treatment (Control). **F** Quantification of Ki67 and GL13 stainings in E. **G** Western blotting of cell lysates from C4-2B xenografts in E, using the indicated markers. Densitometry was carried out for all markers. See also Fig. S8B. **H** q-PCR for GATA2 levels in cell lysates from C4-2B xenografts in E. Magnification: 200x (Objective 20x), scale bars: 30 μm. Inset magnification: 400x (Objective 40x). *P < *0.05* and ***P < *0.001*, of Student’s t-test; n.s., non-significant. Error bars indicate s.e.m. Data shown are representative of at least 3 biological experiments (n ≥ 3)

Based on our in vitro observations and supportive computational analyses regarding an enzalutamide-mediated regulation of senescence and EMT in prostate cancer cells, we asked to recapitulate our key findings in an in vivo setting. Therefore, we performed subcutaneous injections of LNCaP and C4-2B cells in immunodeficient SCID mice, which upon successful tumor formation received or not treatment with enzalutamide (10 mg/kg) for an overall period of 8 days. In keeping with our in vitro findings, enzalutamide-treated LNCaP xenografts displayed increased senescence and reduced proliferation compared to untreated counterparts (Fig. 6A and B). Further analysis of LNCaP cell lysates from extracted tumors showed a marked reduction in CDC6 protein levels upon enzalutamide treatment, accompanied by p21^WAF1/Cip1^ upregulation (Fig. 6C), while GATA2 levels were significantly increased (Fig. 6D), in complete alignment with our in vitro observations. Moreover, EMT was found attenuated in enzalutamide-treated LNCaP xenografts (Fig. 6C, S8A and 8C). In contrast, C4-2B tumors displayed no evidence of senescence regardless of treatment (Fig. 6E-G), combined with CDC6 and EMT activation, as well as increased invasiveness (Fig. 6G, S8B-D), while GATA2 levels were decreased (Fig. 6H). Those results corroborate our previous findings indicating a senescence-inducing role of enzalutamide in responsive cells through GATA2^high^-CDC6^low^ signaling, whereas resistant cells not only continue to display lack of senescence following treatment, but additionally exhibit an enhanced invasive profile accompanied by an opposite GATA2-CDC6 regulation pattern.

## Discussion

**Fig. 7:**
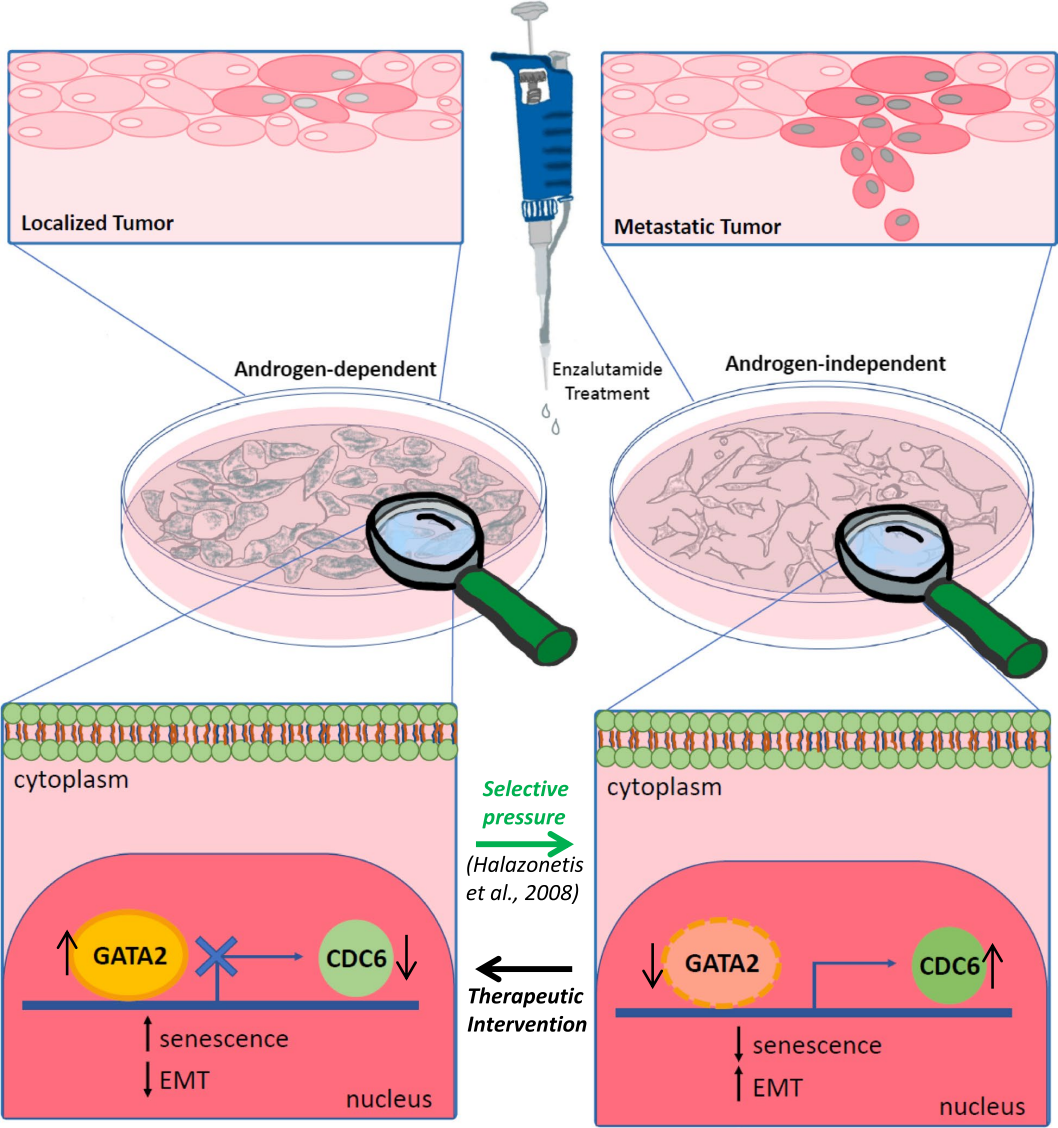
Model. In enzalutamide-sensitive prostate cancers (LNCaP cells), ezalutamide treatment may activate the transcription factor GATA2 which functions as a CDC6 repressor, thereby stimulating cellular senescence at the expense of EMT. However, upon acquiring resistance to enzalutamide (C4-2B and PC-3 cells), prostate tumors exhibit an inverse regulation of the GATA2-CDC6 axis, leading to CDC6 stabilization via endogenous GATA2 downregulation, which activates EMT and abrogates senescence. Bypassing the GATA2-CDC6 axis by direct CDC6 depletion reverses oncogenic features and establishes senescence in enzalutamide-resistant cells, identifying CDC6 as a critical regulator of prostate cancer cell fate and response to therapy

In this study we identify a novel senescence-regulating GATA2-CDC6 signaling axis, differentially modulated in androgen-sensitive and androgen-resistant prostate cancer cells (Fig. 7). We demonstrate that the AR inhibitor enzalutamide triggers GATA2 expression in responsive cells, leading to senescence induction and decreased oncogenic proliferation and invasiveness through direct CDC6 downregulation. On the contrary, androgen-resistant prostate cancer cells display an inverse regulation of the GATA2-CDC6 axis upon treatment, leading to GATA2 reduction and CDC6-driven oncogenic growth and EMT, as well as failure to establish senescence. Importantly, we demonstrate that CDC6 loss is sufficient to reverse oncogenic features and induce senescence even in metastatic, therapy-resistant cells, thereby delineating a key molecular mechanism contributing to progression from localized to metastatic prostate cancer. Moreover, we provide evidence that GATA2-mediated CDC6 expression may be responsible for previously underappreciated detrimental effects on prostate cancer patients starting to acquire resistance to AR inhibitors, thus outlining the clinical setting where anti-AR treatment may constitute a productive therapeutic strategy.

ADT and AR blockade remain the mainstay of therapy for prostate cancer patients, albeit being effective only in the early stages of disease [9]. Enzalutamide is an orally administered second-generation AR inhibitor and a number of clinical trials have shown that it may associate with improved overall survival rates in CRPC patients [50, 51]. Unfortunately, enzalutamide is not effective for all CRPC patients, as there is a portion of those patients who do not respond at all to the drug [52]. Additionally, there are also patients for which the benefits of their initial response to enzalutamide are soon overarched by the acquisition of drug resistance [52], therefore enzalutamide is currently not being used as a first line therapy in metastatic CRPC. On the contrary, recent clinical data have demonstrated that enzalutamide may be considerably more beneficial as first line treatment for hormone-sensitive prostate cancer patients [53]. Our results are in line with those clinical observations, providing a mechanistic explanation behind enzalutamide efficiency in androgen-sensitive prostate patients versus its inefficiency in a large portion of CRPC patients. Our study additionally implies that prolonged treatment of CRPC patients with enzalutamide may increase tumorigenicity upon acquiring resistance, through our identified GATA2-CDC6 axis, potentially justifying the comparatively limited gain in overall survival [54].

The *GATA* family consists of evolutionarily conserved genes encoding pioneer transcription factors, actively regulating the development and differentiation of various tissues [55]. The contribution of GATA2 to prostate cancer tumorigenesis is of increasing interest, as GATA2 is a major determinant of urogenital development [55]. In prostate cancer, GATA2 has been recently shown to promote AR signaling [56], an inducer of *CDC6* transcription [16, 57]. Our model displays a GATA2-mediated suppression of CDC6, where a direct GATA2-dependent CDC6 regulation may be more dominant over AR. Indeed, GATA2 depletion is sufficient to reverse CDC6 reduction conferred by AR inhibition, as clearly shown in androgen-sensitive cells (Fig. 2B and C). However, it still remains unknown why GATA2 levels are reciprocally regulated in androgen-sensitive and –resistant cells upon enzalutamide treatment. Upstream regulators of GATA2 have been identified in prostate cancer, such as the E3 ligase COP1, which was found to elicit GATA2 ubiquitination and its subsequent degradation [56]. The transcription factor FOXA1 was also found to act upstream of GATA2 in prostate cancer, recruiting it to Forkhead DNA Binding Domain (FKHD)-containing genomic sites [58]. Interestingly, the GATA2 cistrome in CRPC was found to significantly overlap with bromodomain and extraterminal (BET) proteins, while BET inhibitors compromized GATA2 activity [59]. Although those potential regulators of GATA2 may be involved in the differential response to enzalutamide treatment, the exact mechanism remains to be determined.

Several molecular players have been found to confer survival to prostate cancer cells by bypassing the AR pathway, rendering it non-essential for cellular growth. For instance, upregulation of the *VAV3* and *TWIST1* oncogenes or repression of the DKK3 tumor suppressor have been linked with prostate cancer progression independent of AR signaling [60]. Kinase-dependent signaling pathways may additionally become activated as a bypass mechanism to AR signaling [61]. Using *Pten* conditional prostate deletion mice it was shown that *Pten* loss, which leads to higher PI3K activity, may render prostate cancer cells proliferative even in the absence of androgen, thereby circumventing dependency on AR [62]. Moreover, activation of the glucocorticoid receptor (GR) has been observed upon AR inactivation, likely conferring a survival benefit to prostate cancer cells progressing into castration resistance [63, 64].

It is widely accepted that cellular senescence is a tumor barrier mechanism triggered by a number of stimuli, including oncogene activation [21, 22, 65]. As such, it was found that CDC6 induction may establish senescence through a process termed oncogene-induced senescence (OIS) [19, 21]. Our data shed light on a novel aspect of CDC6-mediated senescence, where loss of CDC6 rather than overexpression may immediately establish senescence in prostate cancer. In our dataset, CDC6-mediated senescence was directly elicited both after enzalutamide treatment in androgen-sensitive cells and RNAi-mediated CDC6 silencing in CRPC cells. A similar effect was also observed in nasopharyngeal carcinoma cells where CDC6 depletion led to irradiation-mediated senescence [20]. On the other hand, we show that upregulation of CDC6 upon acquired drug resistance not only fails to establish senescence, but is also accompanied by increased EMT. This could be explained by the observation that prolonged expression of oncogenes such as CDC6 may ultimately elicit the process of “escape” from senescence and cell cycle re-entry, resulting in the acquisition of even more aggressive oncogenic features [12, 25, 66], driven by distinct genetic and epigenetic alterations [23]. Indeed, ADT-induced senescence in androgen-sensitive prostate cancer cells was found to be permissive for progression into CRPC, due to senescent cell subpopulations reacquiring proliferative capacity [13].

An important point regarding acquired resistance to therapy in prostate cancer revolves around deciphering the mechanism underlying the transition of cells from a responsive to an irresponsive state. According to our model, treatment of androgen-sensitive cells with AR inhibitors leads to CDC6 downregulation; this, however, may be responsible for DNA under-replication, potentially leading to DNA damage as cells may enter mitosis with incompletely replicated DNA or unresolved chromosomes [67, 68]. DNA under-replication becomes permissive for gradual accumulation of genomic instability, which eventually inactivates tumor suppressive mechanisms as a result of selective pressure [19, 21, 22, 25, 69]. The GATA2-CDC6 axis, which functions as such a tumor suppressive mechanism in an androgen-sensitive environment may, therefore, be rewired to contribute to oncogenic progression.

Several signaling pathways have been found to be implicated in prostate tumorigenesis, potentially contributing to the regulation of senescence [70–73]. Our work demonstrates that abrogating CDC6 expression either through GATA2 or via direct CDC6 inhibition may establish senescence in both androgen-sensitive and –resistant prostate cancer cells. Thus, the identified senescence-modulating GATA2-CDC6 axis offers a therapeutic window, which could be readily exploited by the rapidly developing field of senolytics aimed at selectively eliminating senescent cells in a tissue [74–76]. Numerous chemical compounds are currently being investigated as potential senolytic drugs [76–78], while several natural compounds exerting important protective or anti-cancer effects are also tested as anti-senescence agents [79–81].

## Conclusion

Our study identifies the GATA2-CDC6 axis as a crucial regulator of cellular senescence and EMT in prostate cancer cells, clarifying the clinical setting where treatment with AR inhibitors may be beneficial for patients, or instead, adding to their oncogenic load. Our findings may have immediate therapeutic implications, as direct modulation of the GATA2-CDC6 axis may dramatically alter prostate cancer cell fate and unlock additional treatment options.

## Supplementary Information

**Additional file 1: Figure S1.** SA-β-gal staining confirms the senescence phenotype of enzalutamide-sensitive and -resistant cells. A SA-β-gal staining of LNCaP cells upon enzalutamide treatment compared to Control. B Quantification of SA-β-gal stainings in A. C Same as A, for C4-2B cells. D Quantification of SA-β-gal stainings in C. E SA-β-gal stainings of C4-2B cells of Fig. 3E. F Quantification of SA-β-gal stainings in E. Magnification: 100x (Objective 10x), scale bars: 60 μm. Inset magnification: 400x (Objective 40x). Enzalutamide was used at a 10 μM concentration. ***P < *0.001*, of Student’s t-test; n.s., non-significant. Error bars indicate s.e.m. Data shown are representative of at least 3 biological experiments (n ≥ 3). **Figure S2.** Densitometry accompanying key observations acquired by Western blotting. A Densitomery of indicated markers over control (GAPDH) on immunoblotting presented in Fig. 2B. B Same as A, for Fig. 3A. C Densitometry of indicated markers over control (GAPDH) for immunoblotting in C4-2B cells of Fig. 3D. D Same as C, for PC-3 cells of Fig. 3D. **P < *0.001* and ***P < *0.001*, of Student’s t-test (comparing individual lanes to lane 1); n.s., non-significant. Error bars indicate s.e.m. Data shown are representative of at least 3 biological experiments (n ≥ 3). **Figure S3.** Loss of CDC6 establishes senescence in enzalutamide- resistant PC-3 cells. A Immunofluorescence for dual GL13/p21^WAF1/Cip1^ staining and B quantification confirms senescence induction only in CDC6-depleted PC-3 cells. C SA-β-gal staining of PC-3 cells in A and D quantification of SA-B-gal stainings confirm senescence induction upon CDC6 depletion. Magnification: 100x (Objective 10x), scale bars: 60 μm. Inset magnification: 400x (Objective 40x). Enzalutamide was used at a 10 μM concentration. *P < *0.05* and ***P < *0.001*, of Student’s t-test; n.s., non-significant. Error bars indicate s.e.m. Data shown are representative of at least 3 biological experiments (n ≥ 3). **Figure S4.** CDC6 loss decreases proliferation of enzalutamide-resistant cells regardless of treatment. A Immunocytochemistry for Ki67 levels in C4-2B cells from Fig. 3D and B quantification of Ki67-positive cells displaying reduced proliferation upon CDC6 loss. Magnification: 100x (Objective 10x), scale bars: 60 μm. Inset magnification: 400x (Objective 40x). C Same as A, for PC-3 cells from Fig. 3D. Magnification: 200x (Objective 20x), scale bars: 30 μm. D Same as B, for PC-3 cells from Fig. 3D. E qPCR for EMT markers *CDH1*, *ZEB1* and *SNAI1* in the indicated conditions of PC-3 cells from Fig. 3D. Data are normalized to GAPDH expression. Enzalutamide was used at a 10 μM concentration. **P < *0.01* and ***P < *0.001*, of Student’s t-test; n.s., non-significant. Error bars indicate s.e.m. Data shown are representative of at least 3 biological experiments (n ≥ 3). **Figure S5.** Enzalutamide treatment fails to induce senescence in prostate cancer cells acquiring resistance. A GL13 staining assessed by immunofluorescence in enzalutamide-resistant C4-2B cells from Fig. 4A displays no induction of senescence in cells receiving further treatment. B Quantification of positive GL13 cells in A. C Immunocytochemistry for GL13 levels in PC-3 cells from Fig. 4A indicating no senescence induction in cells receiving further treatment. Magnification: 200x (Objective 20x), scale bars: 30 μm. D Quantification of GL13-positive PC-3 cells in C. E SA-β-gal staining in PC-3 cells from Fig. 4A confirming the GL13 staining. Magnification: 100x (Objective 10x), scale bars: 60 μm. Inset magnification: 400x (Objective 40x). F Quantification of SA-β-gal-positive cells in E. G Immunohistochemistry for Ki67 levels in C4-2B cells from Fig. 4A. Magnification: 100x (Objective 10x), scale bars: 60 μm. Inset magnification: 400x (Objective 40x). H Quantification of Ki67-positive cells in G. Enzalutamide was used at a 10 μM concentration. N.s., non-significant. Error bars indicate s.e.m. Data shown are representative of at least 3 biological experiments (n ≥ 3). **Figure S6.** Androgen-resistant prostate cancer cells display increased in vitro migration and invasion in response to enzalutamide. A Wound healing assay of control or enzalutamide-treated PC-3 cells, demonstrating migration at the indicated time points post wound formation. The cells were subjected to the assay having already received or not enzalutamide for 72 h. The dashed lines indicate the wound borders in each case. B Quantification of migration in A. C Quantification of invasion of control or enzalutamide-treated PC-3 cells, using a colorimetric invasion assay. The cells were subjected to the assay having already received or not enzalutamide for 72 h. Enzalutamide was used at a 10 μM concentration. Magnification: 50x (Objective 5x), scale bars: 200 μm. ***P < *0.001*, of Student’s t-test; n.s., non-significant. Error bars indicate s.e.m. Data shown are representative of at least 3 biological experiments (n ≥ 3). **Figure S7.** Enzalutamide treatment is not accompanied by autophagy or apoptosis in either enzalutamide-sensitive or -resistant prostate cancer cells. A Electron micrographs of control or enzalutamide-treated LNCaP and C4-2B cells showing no autophagy activation following treatment. Autophagic vacuoles were not observed in neither treated nor untreated cells. N: nucleus; m: mitochondrion. Scale bars for LNCaP cells: 1 μm; for C4-2B cells: 500 nm. B Immunocytochemistry for cleaved caspase-3 staining in LNCaP, C4-2B and PC-3 control or enzalutamide-treated cells displaying no induction of apoptosis. PC-3 cells subjected to 5% DMSO treatment for 24 h were used as positive cleaved caspase-3 control. Magnification: 200x (Objective 20x), scale bars: 30 μm. C Quantification of cleaved caspase-3 stainings in B. Enzalutamide was used at a 10 μΜ concentration. Statistics were determined using Student’s t test; Ν.s., non-significant. Error bars indicate s.e.m. Data shown are representative of at least 3 biological experiments (n ≥ 3). **Figure S8.** Enzalutamide treatment confers opposite regulation of EMT markers between responsive and resistant cells in vivo. A qPCR for EMT markers *CDH1*, *ZEB1* and *SNAI1* in LNCaP mouse xenografts treated or not with enzalutamide (10 mg/kg). Enzalutamide treatment is accompanied by EMT attenuation. B Same as A in C4-2B mouse xenografts. Enzalutamide treatment is accompanied by pronounced EMT compared to control. C Τumor volume curves over days of treatment in mice injected with the indicated cells. D Immunohistochemistry of extracted mouse tumors confirming increased invasiveness of C4-2B cells receiving enzalutamide treatment versus control, into the underlying muscle tissue. Magnification: 200x (Objective 20x), scale bars: 30 μm. **P < *0.01* and ***P < *0.001*, of Student’s t-test. Error bars indicate s.e.m. Data shown are representative of at least 3 biological experiments (n ≥ 3).

**Additional file 2: Table S1.** RNA sequencing raw data and analysis of enzalutamide-treated LNCaP cells versus untreated counterparts.

**Additional file 3: Table S2.** Pathway expression analysis of in vitro and in vivo enzalutamide-sensitive and –resistant prostate cancer samples. RNA sequencing data were retrieved from publically available datasets with accession numbers GSE55030, GSE109708, GSE184168, GSE163240, GSE150807 and GSE151083.

## Data Availability

The datasets used and/or analyzed during the current study are available from the corresponding author on reasonable request.
