# Supplementary figures and images for: A GATA2-CDC6 axis modulates androgen receptor blockade-induced senescence in prostate cancer
Source: J Exp Clin Cancer Res. 2023 Jul 29;42:187. doi: 10.1186/s13046-023-02769-z (PMC10386253; doi:10.1186/s13046-023-02769-z)

Figure S1

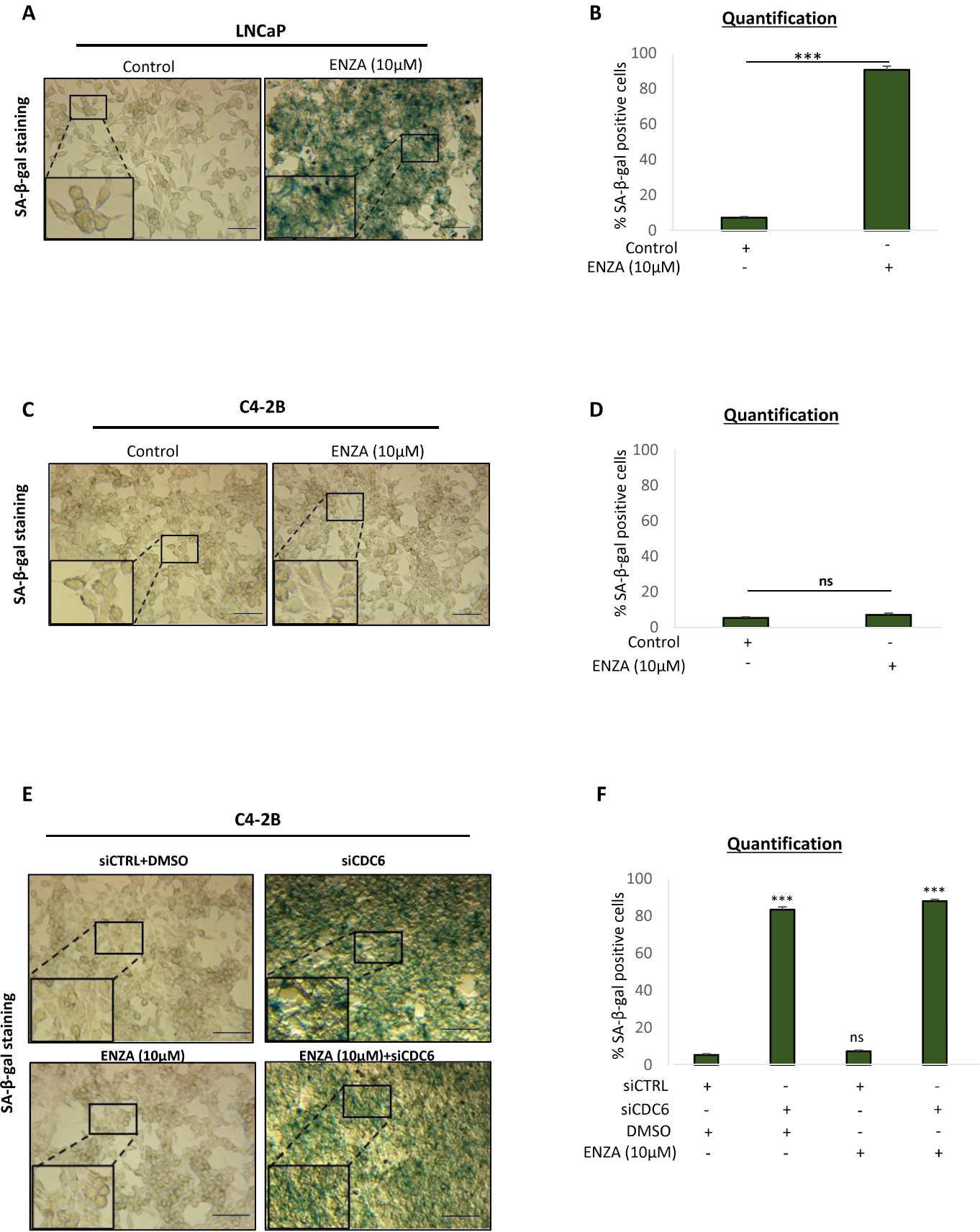

Figure S2

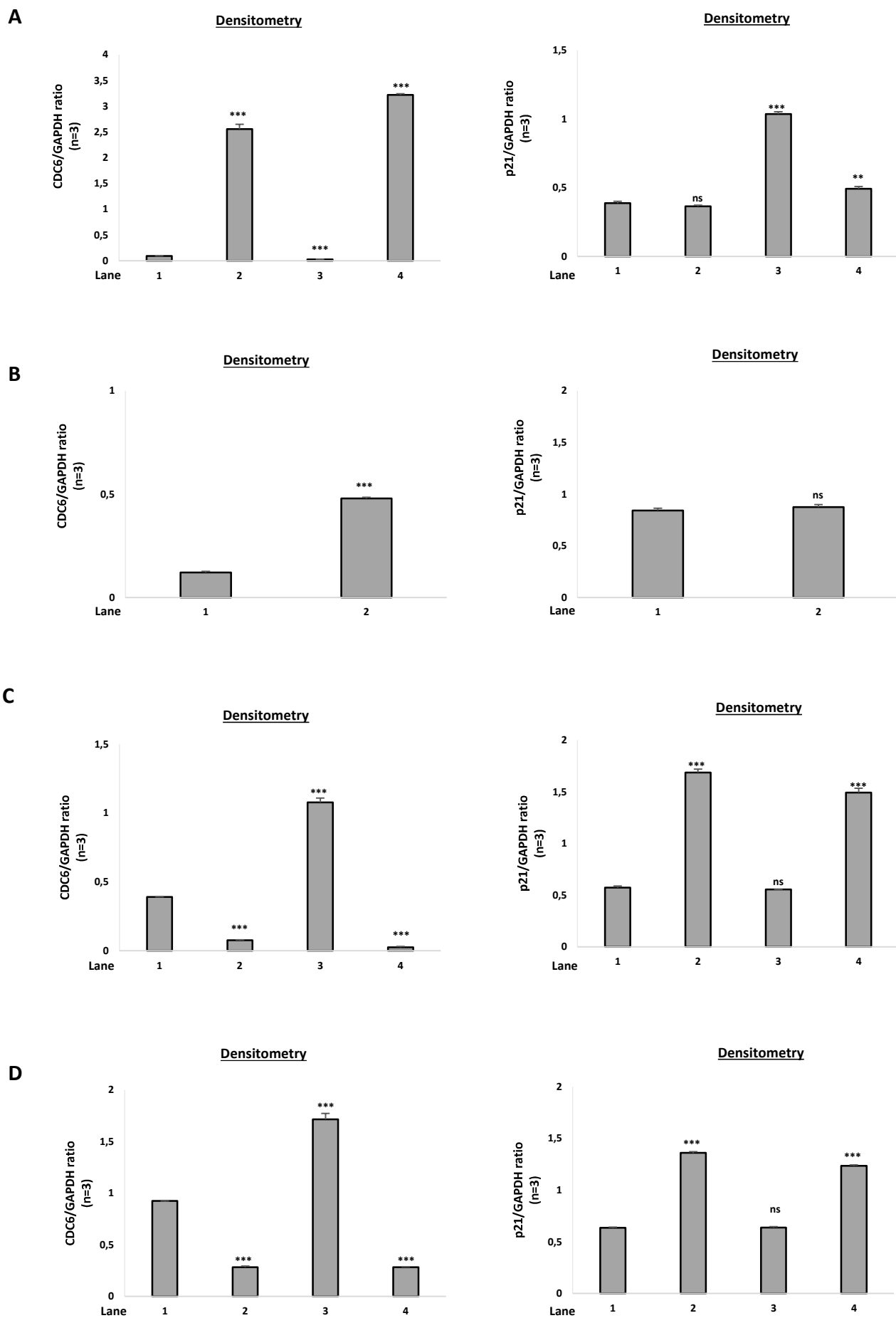

Figure S3

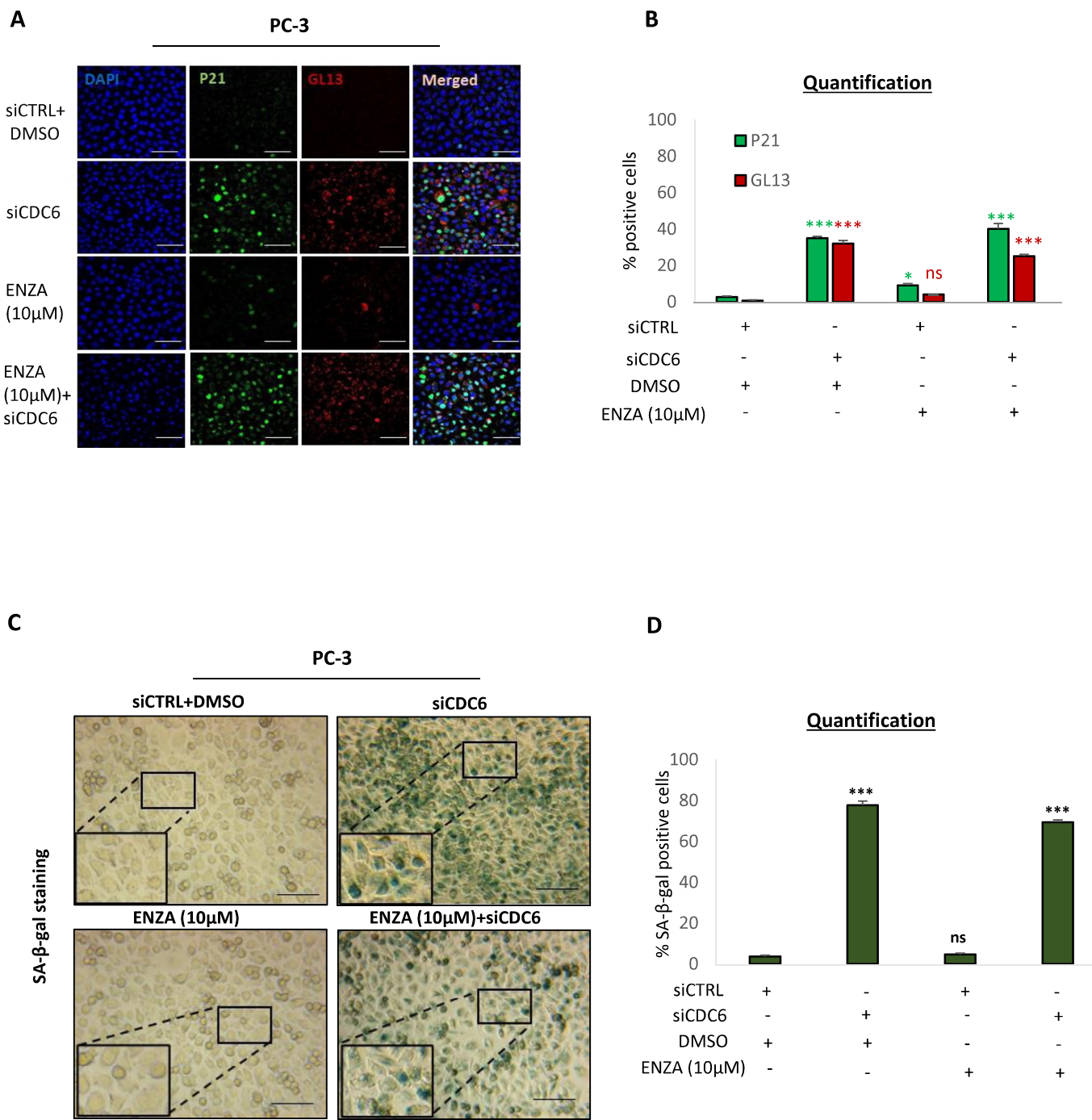

Figure S4

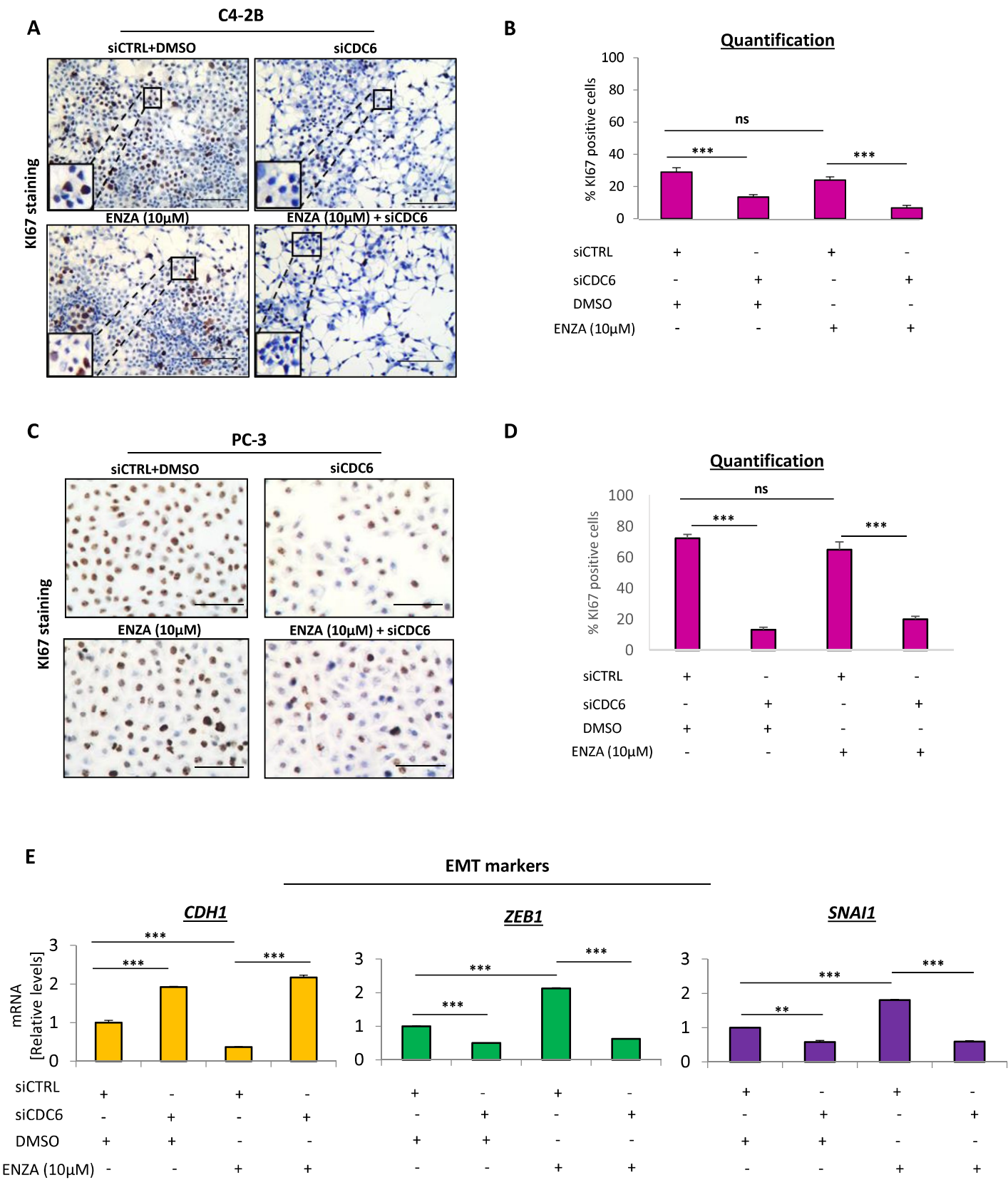

Figure S5

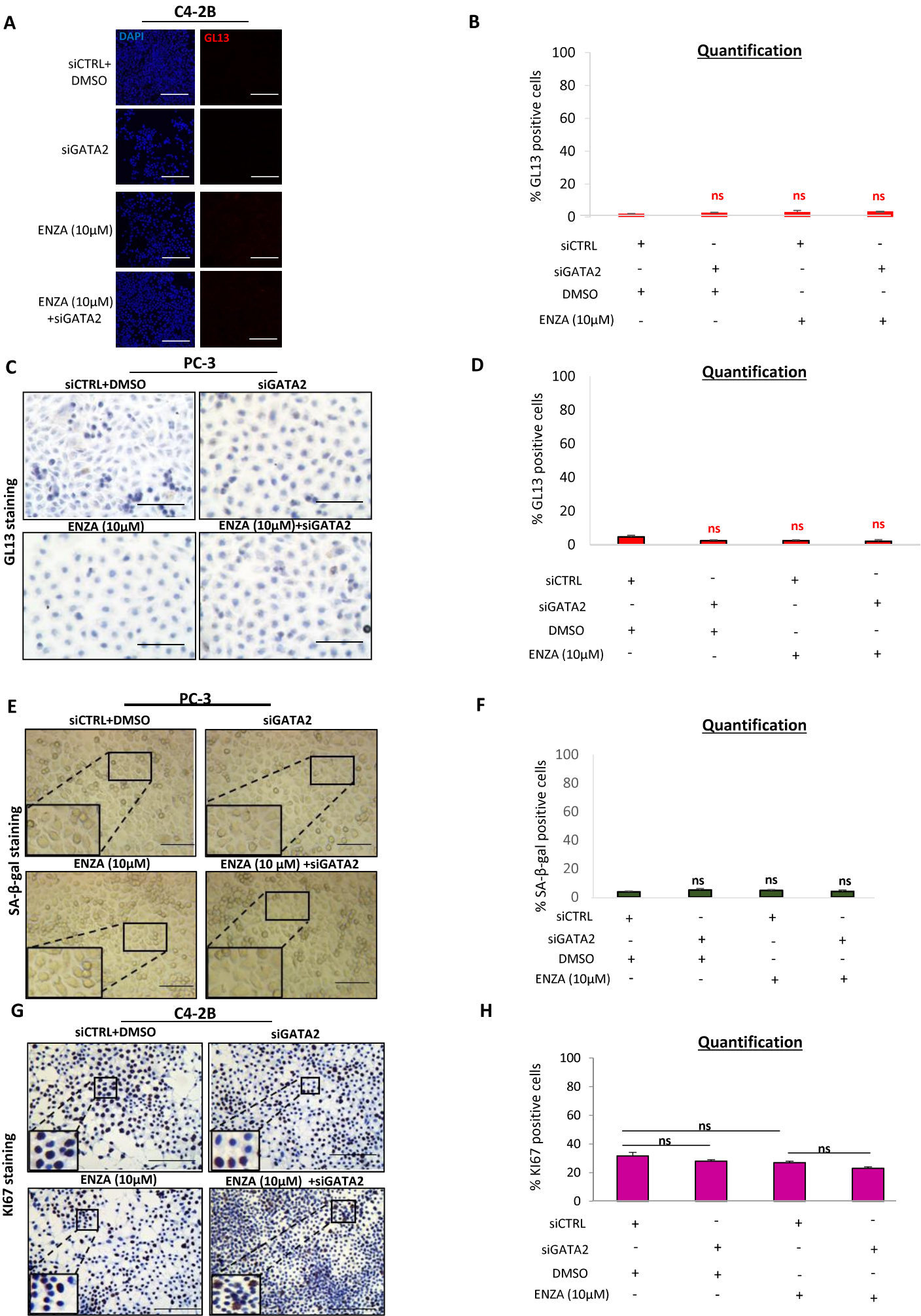

Figure S6

A

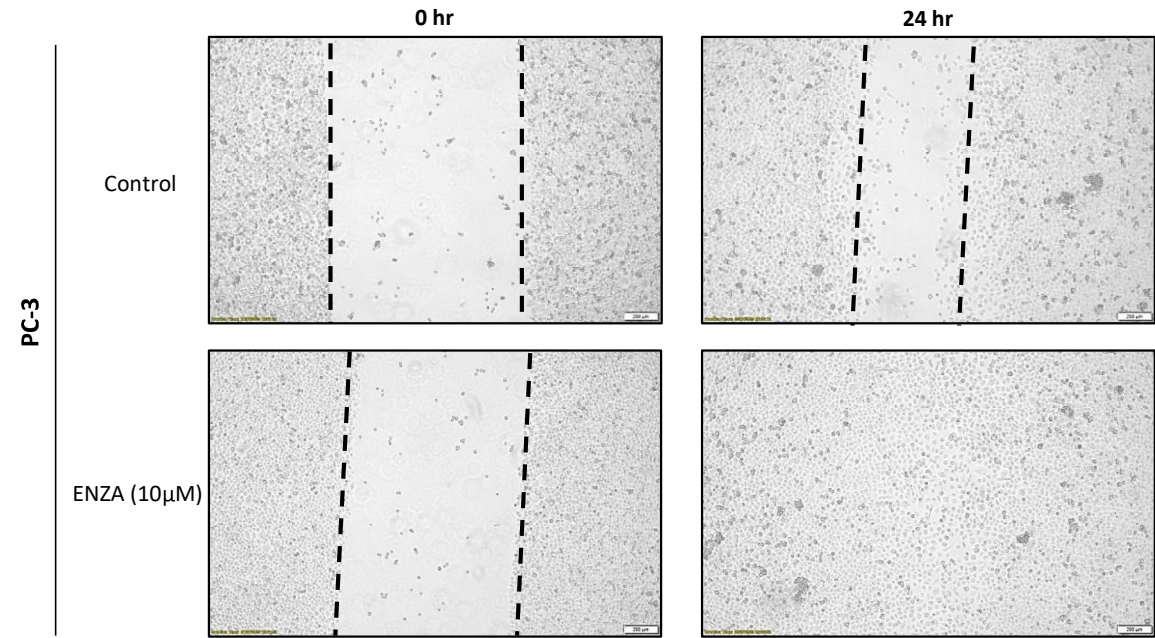

B

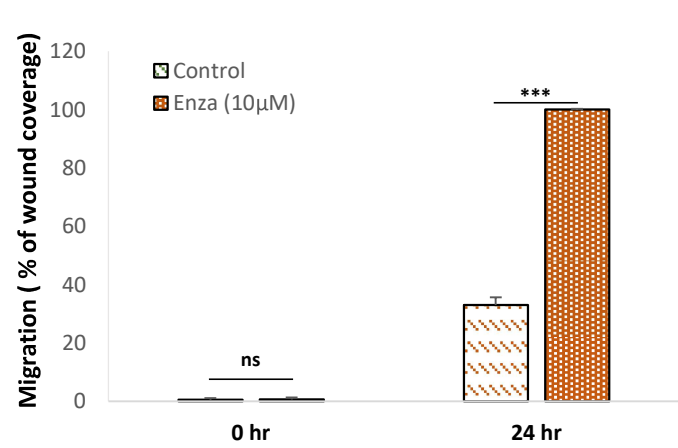

C

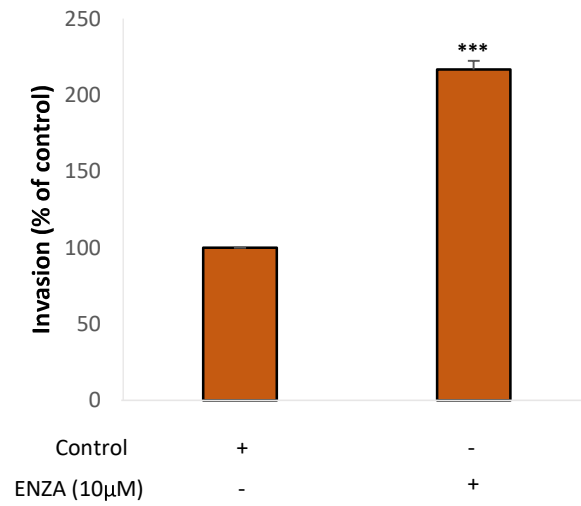

Figure S7

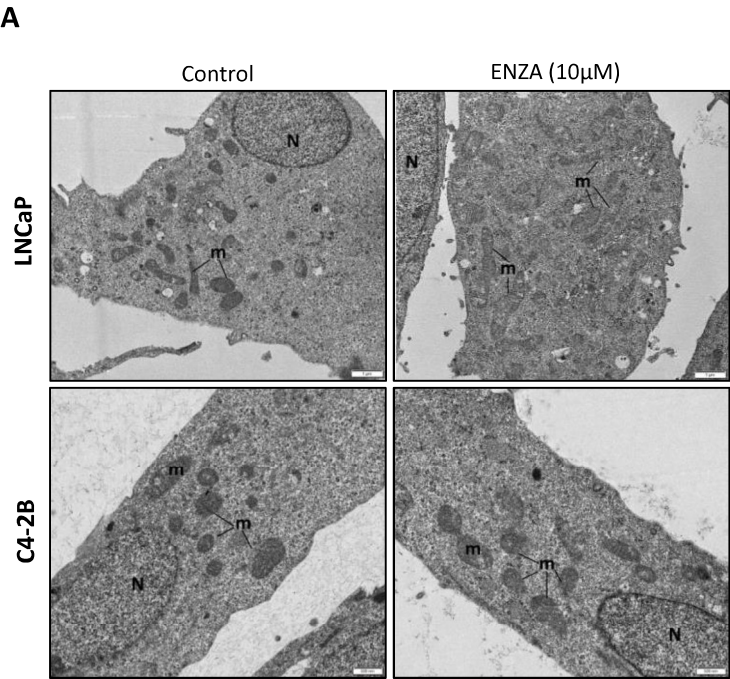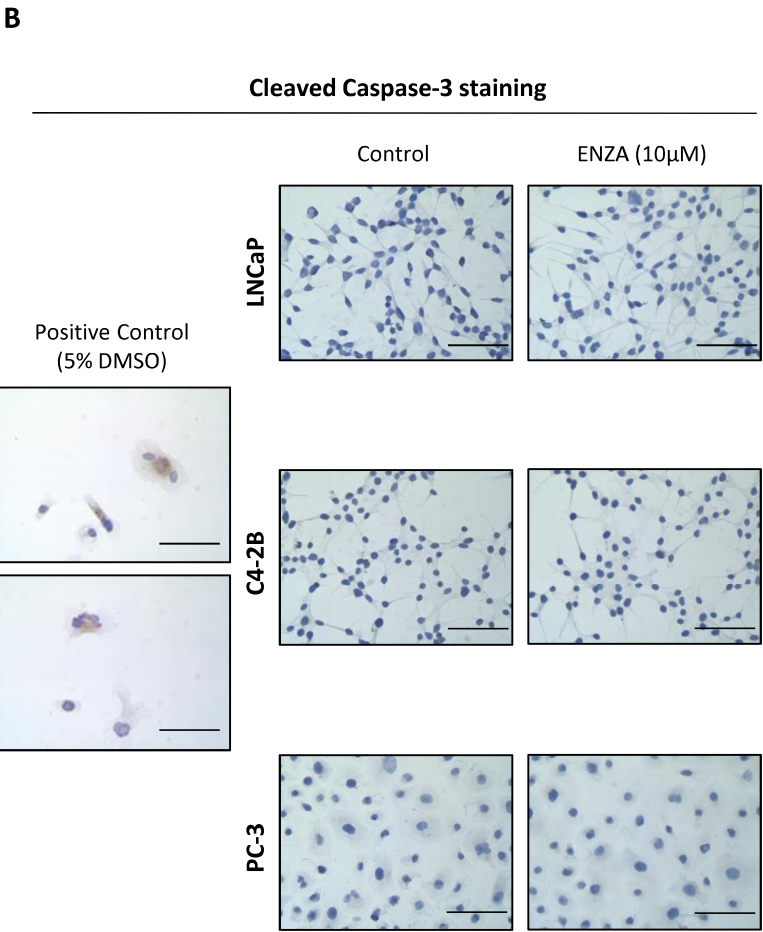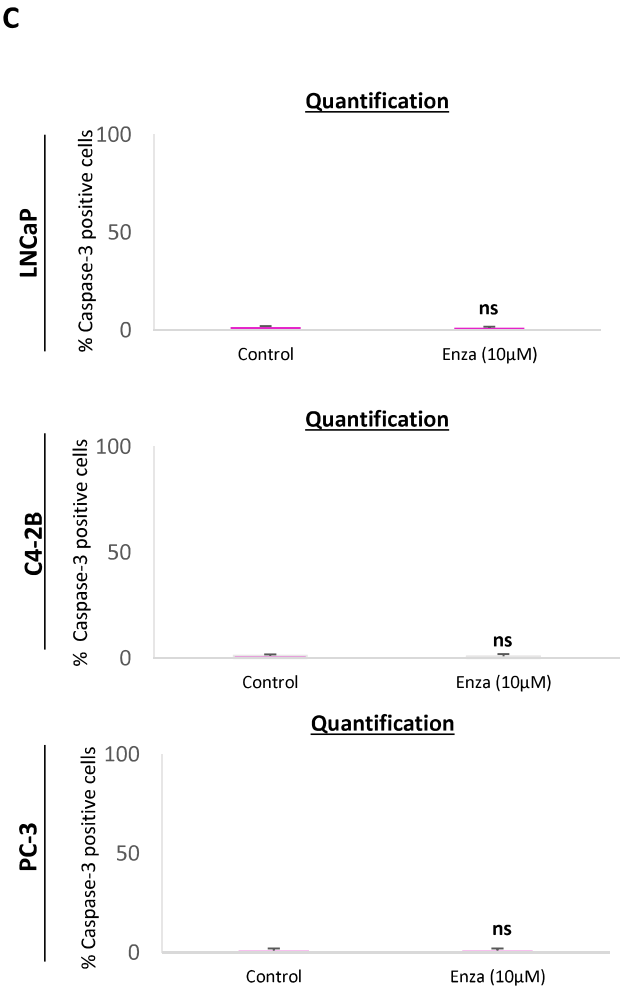

Figure S8

A

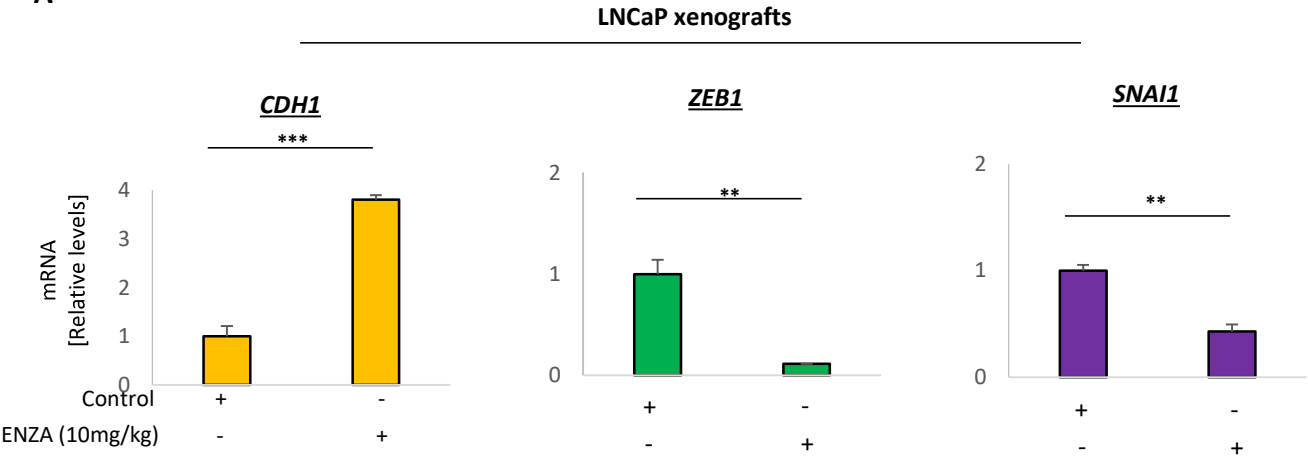

B

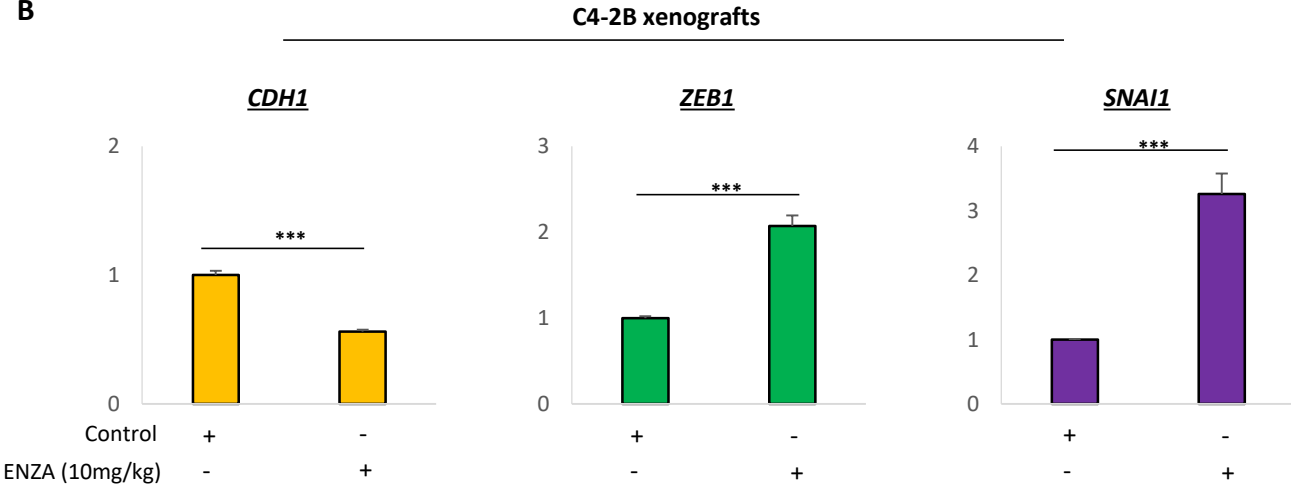

C

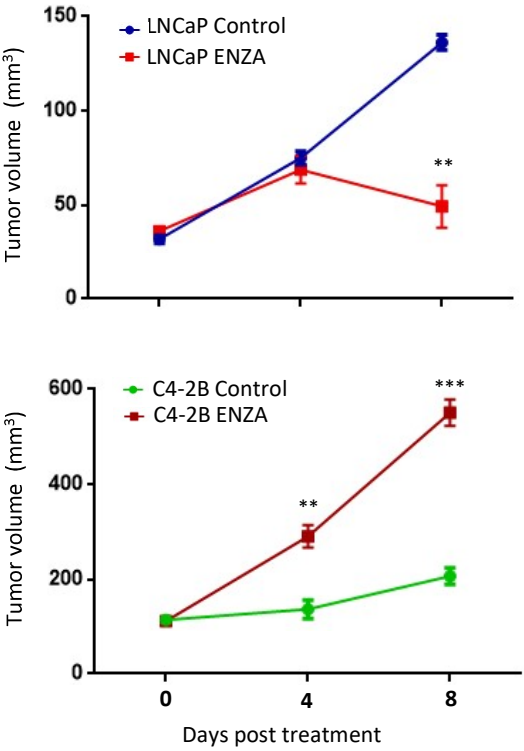

D

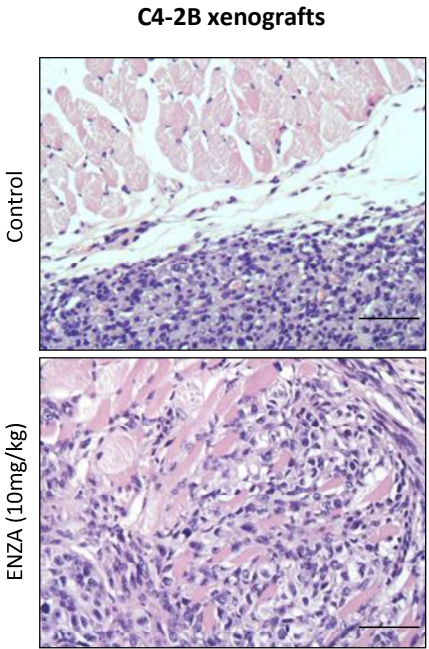

Supplement: Supplementary file 1 — Additional file 1: Figure S1. SA-β-gal staining confirms the senescence phenotype of enzalutamide-sensitive and -resistant cells. A SA-β-gal staining of LNCaP cells upon enzalutamide treatment compared to Control. B Quantification of SA-β-gal stainings in A. C Same as A, for C4-2B cells. D Quantification of SA-β-gal stainings in C. E SA-β-gal stainings of C4-2B cells of Fig. 3E. F Quantification of SA-β-gal stainings in E. Magnification: 100x (Objective 10x), scale bars: 60 μm. Inset magnification: 400x (Objective 40x). Enzalutamide was used at a 10 μM concentration. ***P < 0.001, of Student’s t-test; n.s., non-significant. Error bars indicate s.e.m. Data shown are representative of at least 3 biological experiments (n ≥ 3). Figure S2. Densitometry accompanying key observations acquired by Western blotting. A Densitomery of indicated markers over control (GAPDH) on immunoblotting presented in Fig. 2B. B Same as A, for Fig. 3A. C Densitometry of indicated markers over control (GAPDH) for immunoblotting in C4-2B cells of Fig. 3D. D Same as C, for PC-3 cells of Fig. 3D. **P < 0.001 and ***P < 0.001, of Student’s t-test (comparing individual lanes to lane 1); n.s., non-significant. Error bars indicate s.e.m. Data shown are representative of at least 3 biological experiments (n ≥ 3). Figure S3. Loss of CDC6 establishes senescence in enzalutamide- resistant PC-3 cells. A Immunofluorescence for dual GL13/p21WAF1/Cip1 staining and B quantification confirms senescence induction only in CDC6-depleted PC-3 cells. C SA-β-gal staining of PC-3 cells in A and D quantification of SA-B-gal stainings confirm senescence induction upon CDC6 depletion. Magnification: 100x (Objective 10x), scale bars: 60 μm. Inset magnification: 400x (Objective 40x). Enzalutamide was used at a 10 μM concentration. *P < 0.05 and ***P < 0.001, of Student’s t-test; n.s., non-significant. Error bars indicate s.e.m. Data shown are representative of at least 3 biological experiments (n ≥ 3). Figure S4. CDC6 lo [file 13046_2023_2769_MOESM1_ESM.pdf]
